# Conjugation‐Induced Spin Delocalization in Helical Chiral Carbon Radicals via Through‐Bond and Through‐Space Effects

**DOI:** 10.1002/advs.202304563

**Published:** 2023-10-22

**Authors:** Longhui Duan, Xiaoping Xue, Biqiong Hong, Zhenhua Gu

**Affiliations:** ^1^ Hefei National Research Center for Physical Sciences at the Microscale Department of Chemistry University of Science and Technology of China 96 Jinzhai Road Hefei Anhui 230026 P. R. China; ^2^ College of Science Henan Agricultural University Zhengzhou Henan 450002 P. R. China; ^3^ College of Materials and Chemical Engineering Minjiang University Fuzhou Fujian 350108 P. R. China

**Keywords:** cross‐coupling reactions, helical chirality, stable carbon radicals, through‐space delocalization

## Abstract

A class of highly stable hydrocarbon radicals with helical chirality are synthesized, which can be isolated and purified by routine column chromatography on silica gel. These carbon‐centered radicals are stabilized by through‐bond delocalization and intramolecular through‐space conjugation, which is evidenced by Density Functional Theory (DFT) calculation. The high stability enables to directly modify the carbon radical via palladium‐catalyzed cross‐coupling with the radical being untapped. The structures and optoelectronic properties are investigated with a variety of experimental methods, including Electron Paramagnetic Resonance (EPR), Ultraviolet Visisble Near Infrared (UV–vis–NIR) measurements, Cyclic Voltammetry (CV), Thermogravimetry Analysis (TGA), Circular Dichroism (CD) spectra, High‐Performance Liquid Chromatography (HPLC), and X‐ray crystallographic analysis. DFT calculations indicated that the 9‐anthryl helical radical is more stable than its tail‐to‐tail σ‐dimer over 13.2 kJ mol^−1^, which is consistent with experimental observations.

## Graphical abstract

A class of stable carbon radicals that are stabilized by delocalization through both through‐bond and through‐space conjugation, are successfully synthesized. The resulting helical structure not only makes the radicals inherently chiral, but also provides great stability, making them ideal for both optoelectronic property investigation and direct chemical decoration.

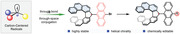

## 1 Introduction

**Figure 1:**
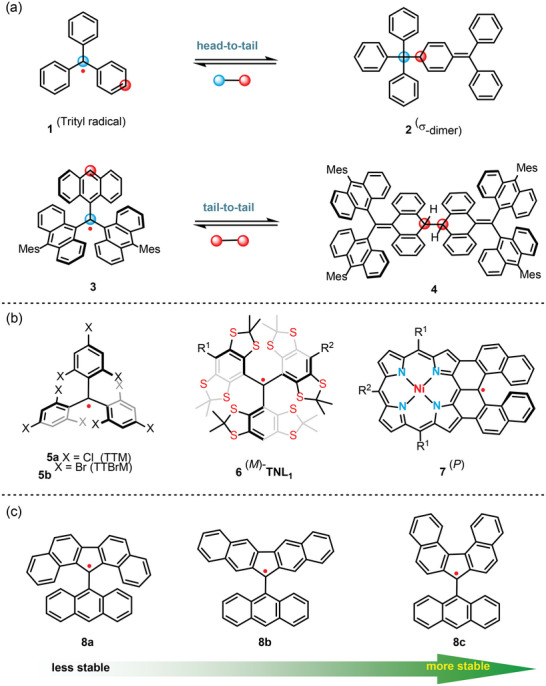
a) Triaryl‐methane radicals and their dimerization. b) Chiral triaryl‐methane radicals. c) Structure‐stability relationship of fluorenyl‐based radicals. It was found that some sterically unprotected hydrocarbon radicals could be stabilized by highly spin‐delocalization through intermolecular π‐dimerization (**9** → **10**), which would give a free radical monomer in solution.^[^ ^13^ ^]^ The large extent of electron delocalization well suppressed σ‐dimerization. Compound **11**, 1,2,3,4,5,6‐hexa[*p*‐(*N,N*‐diethylaminophenyl)]benzene, readily underwent one‐electron oxidation to form stable radical cation **12**, in which extensive through‐space π‐electron delocalization would result in complete toroidal conjugation among the six *p*‐(*N,N*‐diethylamino)phenyl rings (**Figure** **2**a).^[^ ^14^ ^]^ On the other hand, helicenes represent a unique class of nonplanar screw‐like structures, featuring ortho‐fused aromatic rings. They can usually exhibit stable helical chirality and possess an extensively conjugated system.^[^ ^23^, ^28^ ^]^ The distinctive helical skeleton also offers an opportunity for π‐delocalization via through‐space conjugation, resulting in unique optical and electrochemical properties.^[^ ^17^ ^]^ In this work, we have synthesized a class of highly stable carbon‐centered radicals, which were stabilized by through‐bond delocalization and intramolecular through‐space conjugation of the helical skeleton. Notably, no σ‐dimerization was observed regardless of their solid or solution state. The highly stable helical chirality also enabled us to investigate their chiroptical properties. Furthermore, these stable radicals also can be submitted to cross‐coupling reaction with the radical being untapped (Figure 2b).

The synthesis and investigations of optical properties of stable organic radicals have gained significant attention in recent years.^[^ ^1^ ^]^ The unique open‐shell electronic properties owing to an unpaired electron have been widely utilized for exploring novel electronic materials and physiological activity. Nowadays, stable radical compounds have been displaying wide applications in organic functional materials, such as spin materials^[^ ^2^ ^]^ as conducting materials,^[^ ^3^ ^]^ light‐emitting diodes,^[^ ^4^ ^]^ and quantum information science fields,^[^ ^5^ ^]^ etc. In the early stage of radical chemistry, heteroatom‐centered radicals have been studied extensively, while carbon‐centered organic radicals have been less exploited due to their inherent instability. They are generally prone to oxidation or dimerization to form closed‐shell structures (**Figure** **1**a).^[^ ^6^ ^]^ In addition, tedious synthetic approaches further restrict the availability of these carbon‐centered radicals, resulting in fewer studies and applications. Thus, the development of highly stable carbon‐centered radicals is highly deserved.

**Figure 2:**
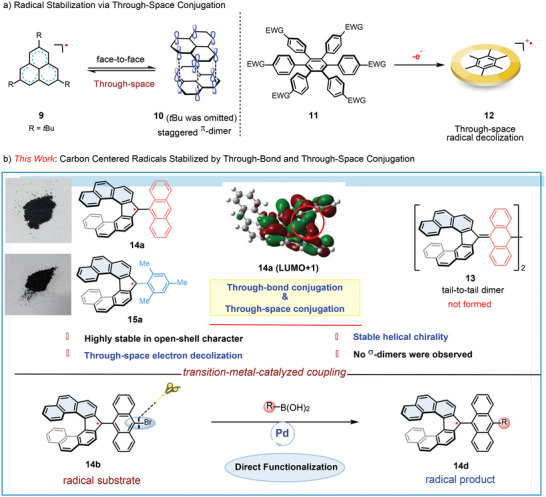
Carbon‐centered radicals stabilized via through‐bond & through‐space conjugation.

Incorporating the spin characteristic of stable radicals with their frameworks' chirality constitutes an appealing approach strategy for acquiring distinctive optoelectronic properties in the realm of material science.^[^ ^7^ ^]^ This approach acknowledges the pivotal role of chirality in determining materials' function and properties, and offers a promising avenue for fabricating bespoke materials with unprecedented applications. In general, controlling the chiral stability of triphenyl‐methane type radicals **1** has always been challenging due to their distinctive propeller configurations and pronounced propensity for σ‐dimerization to yield closed‐shell products, specifically head‐to‐tail dimer **2**.^[^ ^6a^ ^]^ Steric hindrance, which can (partially) restrict the free rotation of functional groups, has traditionally been a primary approach for stabilizing chirality. However, the anthryl radical **3** still tends to form tail‐to‐tail dimer **4** through radical‐radical coupling.^[^ ^6b^ ^]^ It was found that the chloro substituents of tris(2,4,6‐trichlorophenyl)methyl (**TTM**) radical **5a** could effectively inhibit the dimerization of radical–radical coupling (Figure 1b). However, it has a rotational barrier of 19.2 kcal mol^−1^, which racemized easily in the ambient environment.^[^ ^8^ ^]^ Expanding the molecular size or replacing chloride with bromide results in higher chiral stability of **5b** for propeller configurations. In 2011, Marchand–Brynaert and co‐workers found the enantiomers of tetrathiatriarylmethyl radical **6** were configurationally stable enough to be isolated and could be stored for months in the freezer, while it would slowly racemize in solution at room temperature. Introducing inherent chirality represents another effective approach for obtaining stable chiral radicals.^[^ ^9^ ^]^ However, these examples face challenges relating to instability and synthetic difficulty.^[^ ^10^ ^]^ In 2017, Osuka and co‐workers reported an interesting carbon‐centered radical **7** fused with porphyrin‐chelated Ni^II^, which showed high thermal stability, as well as stable chirality (Figure 1b).^[^ ^11^ ^]^ Kubo and co‐workers synthesized three fluorenyl‐based radical isomers (**8a**–**c**), attaching a 9‐anthryl group at the most reactive site of the fluorenyl moiety.^[^ ^12^ ^]^ The 9‐anthryl group stabilized the radicals kinetically, while benzannulation of the fluorene increased the stability of the radicals thermodynamically. Notably, the manner of benzannulation to fluorenyl radical significantly affected its stability, resulting in different half‐lives of **8a**, **8b**, and **8c** in air‐saturated toluene 7, 3.5, and 43 days at room temperature, respectively. However, a strong tendency to form tail‐to‐tail σ‐dimers of **8c** was still observed.

## 2 Results

### 2.1 Synthesis of Helical Radicals

**Scheme 1:**
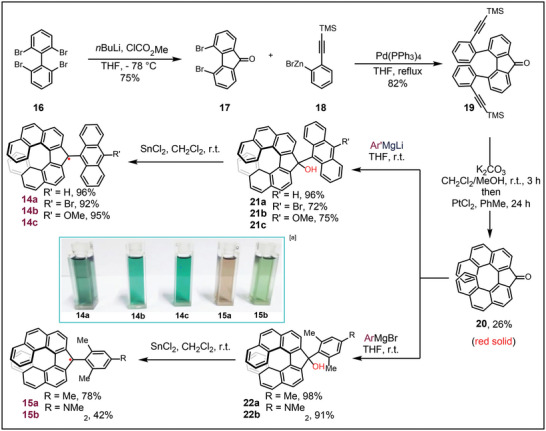
The synthesis of helical radicals. ^[a]^ The solutions dissolved in CHCl_3_ were shown with concentrations of 5 × 10^−2^ mg mL^−1^.

The synthetic route of fluorenyl‐based radicals is shown in **Scheme** **1**. Initially, 2,2′,6,6′‐tetrabromo‐1,1′‐ biphenyl **16** was treated with *n‐*BuLi in THF at −78 °C, followed by the addition of chloroformate, resulting in the formation of 4,5‐dibromo‐9*H*‐fluoren‐9‐one **17** in a good yield. Negishi coupling of **17** with the freshly prepared zinc reagent **18** delivered **19** in 82% yields. Helical ketone **20** could be obtained as a red solid by desilylation of **19**, followed by PtCl_2_‐promoted electrocyclization.^[^ ^18^ ^]^ Upon the Barbier‐type reaction, ketone **20** was smoothly advanced to 9‐aryl‐9*H*‐fluoren‐9‐ols **21a–c** and **22a,b** with different lithium or magnesium reagents, which could be easily reduced by SnCl_2_ to give the target radicals in excellent yields. The reduction of **21a**, **21b,** and **22a** finished within 1 h, while the reduction of electron‐rich **21c** took 5 h. The lowest reaction rate was observed for the reduction of **22b** to **15b**, which took ≈12 h to achieve full conversion. The carbon‐centered radicals can be purified by standard column chromatography on silica gel. The only exception is the slow decomposition of **15b** observed on the column chromatography, which was likely the result of the presence of a dimethylamino group leading to easy oxidation. In contrast to Kubo's results,^[^ ^14^ ^]^ no tail‐to‐tail σ‐dimerization of **14a** was observed, regardless of solution or solid state.

### 2.2 EPR, UV–vis–NIR, and CV Measurements

**Figure 3:**
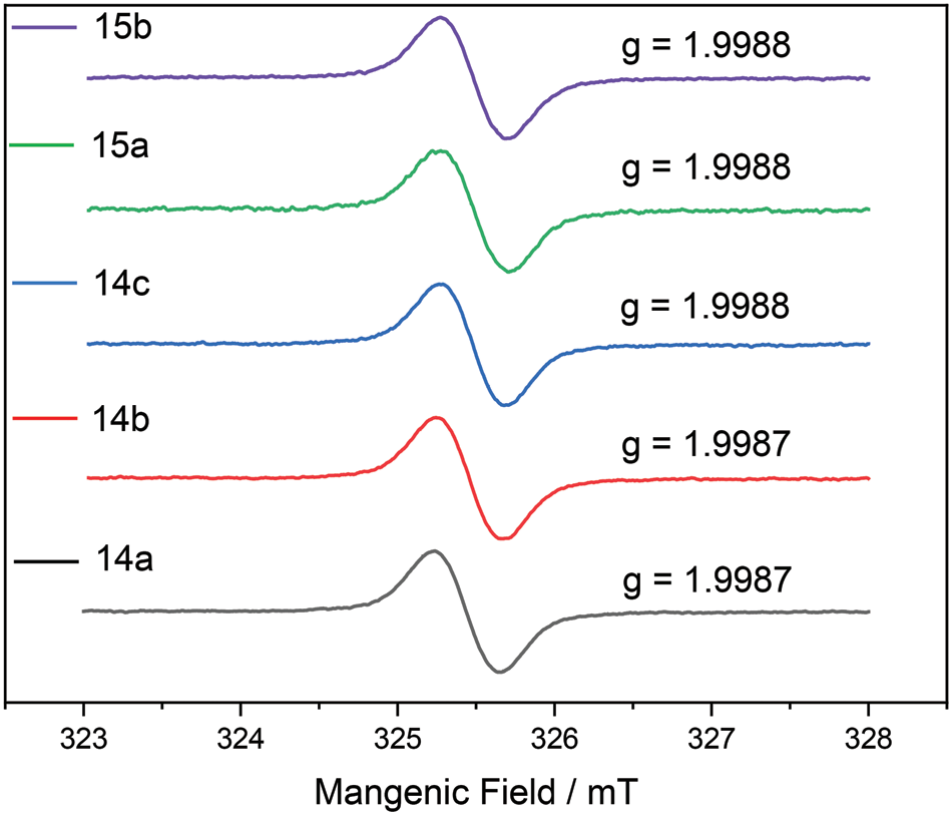
EPR spectra in toluene (*c* = 5 × 10^−5^ m at 298 K).

The EPR experiments were performed in toluene at 298 K, which was unambiguous evidence of the existence of those radicals in the solution state. The strong signals were detected as broad singlet resonances with no discernible hyperfine split, showing *g* values ≈2.000, which were possibly attributed to the good delocalization of radicals over the helical structure (**Figure** **3**).

**Figure 4:**
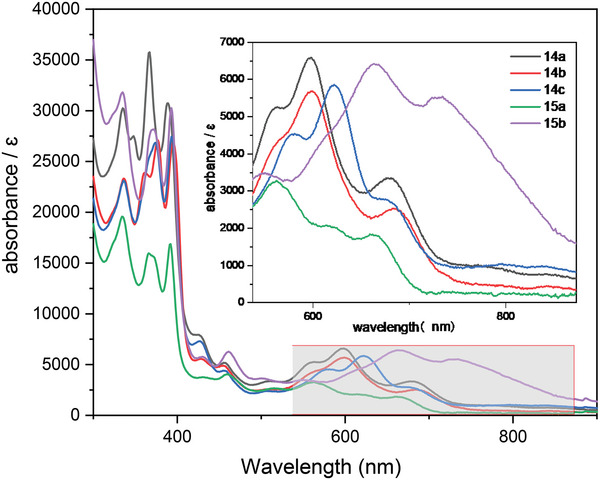
UV–vis–NIR spectra in CH_2_Cl_2_ (*c* = 5 × 10^−5^ m, room temperature), and insert shows a zoom graph for axes magnification of corresponding radicals.

The UV–vis–NIR absorption spectra of these stable radicals were measured in dichloromethane solution at ambient conditions. As is shown in **Figure** **4**, **14a** exhibits three obvious characteristic absorption bonds, which are the main bond (598 nm) induced by the transition from the next highest occupied molecular orbital (HOMO) β‐spin (139B) to single occupied molecular orbital (SOMO) β‐spin (142B), with two shoulders of 563 nm [HOMO β‐spin (38B) to SOMO β‐spin (142B)] and 679 nm [HOMO β‐spin (140B) to SOMO β‐spin (143B)], respectively (for details of the molecular orbital, see Table S10, Supporting Information). A low‐energy transition from the HOMO β‐spin (141B) to SOMO β‐spin (142B) was also predicted with an oscillator strength (*f*) of 0.0013. However, it was not detected due to the low intensity. Similarly, **14b** showed three absorption bonds, a sharp absorption bond with a maximum at 683 nm, along with two bonds at 564, and 599 nm, respectively. A small red‐shift phenomenon was observed in **14c** compared to **14a** and **14b** due to the introduction of a methoxy group at the anthracene ring, which also showed three absorption bonds at 581, 622, and 677 nm. **15a** also exhibits three obvious characteristic absorption bands, which are the main (563 nm), the medium (622 nm), and 665 nm, while **15b** exhibits a massive red‐shift with three obvious characteristic absorption bands, which are the main (665 nm), the medium (731 nm), and 551 nm.

**Figure 5:**
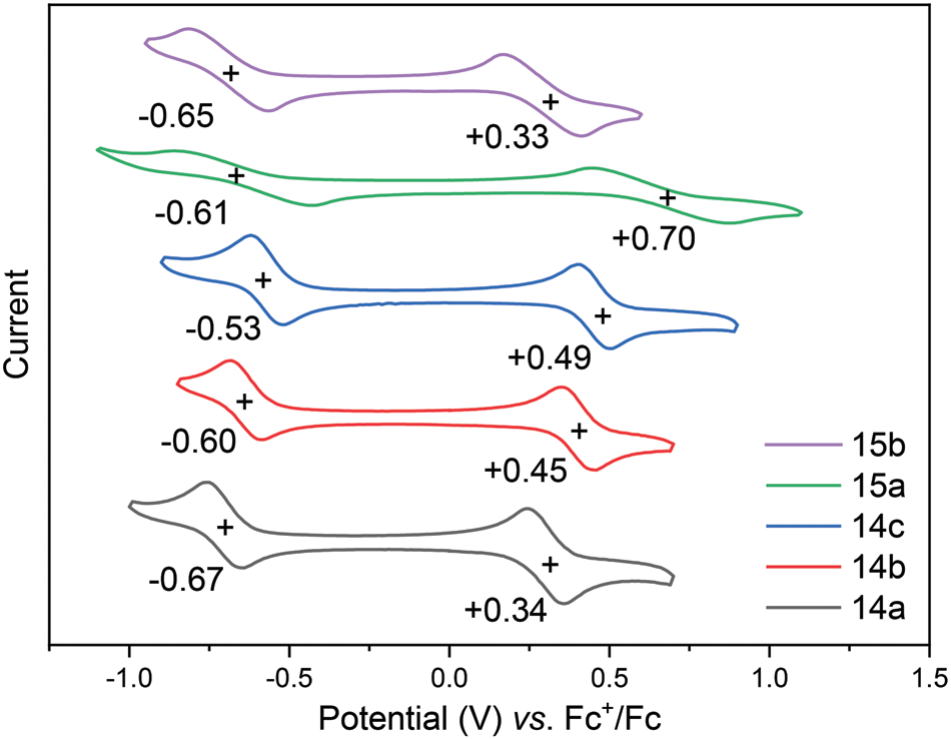
Cyclic voltammograms (V vs Fc/Fc^+^, in 0.1 m *n*Bu_4_NClO_4_ / CH_2_Cl_2_, scan rate 100 mV s^−1^, room temperature).

The electrochemical behavior of these radicals was investigated using CV in a standard three‐electrode electrochemical cell in a solution of tetrabutylammonium hexafluorophosphate (0.1 m) in dry CH_2_Cl_2_ with a scan rate of 100 mV s^−1^. Excellent reversible redox behavior was observed and the half‐wave potentials of the reversible oxidative waves were shown in **Figure** **5**. The values were +0.34, +0.45, and +0.49 V for **14a–c**, and +0.70 and +0.33 V for **15a** and **15b**, respectively, and their half‐wave potentials of the reversible reductive process were −0.67, −0.60, −0.53, −0.61, and −0.65 V, respectively. Notably, radical **15a**, which bears the 2,4,6‐trimethylphenyl group attached to the fluorene structure, showed the highest oxidative half‐wave. On the other hand, replacing the *para* methyl group with *N, N*‐dimethylamino group caused an obvious decrease in oxidative half‐wave, indicating **15b** was easier to be oxidized.

### 2.3 Circular Dichroism Analysis

**Figure 6:**
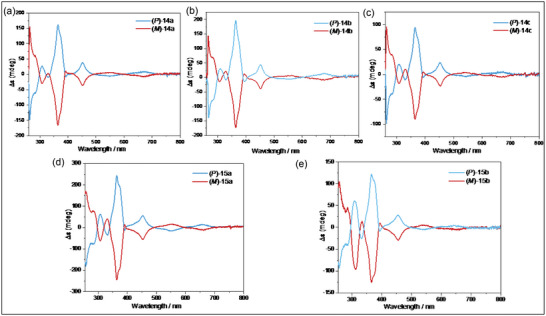
Circular dichroism (CD) spectra of corresponding helical carbon radicals in CH_2_Cl_2_ (*c* = 5 × 10^−5^ m, room temperature).

The helical skeleton not only provided a large π‐system and through‐space conjugation to stabilize the radical, but it also allowed for the formation of stable helical chirality. The resolution of helical cyclopentanone **20** was carried out efficiently with the preparative High‐Performance Liquid Chromatography on a chiral stationary, resulting in two enantiomers that were advanced to optically active radicals **14a–c** and **15a,b**.^[^ ^18^ ^]^ These molecules were characterized using CD analysis in CH_2_Cl_2_ solution, which revealed excellent mirror images to their corresponding enantiomers (**Figure** **6**). The substituents at the anthryl group had minimal effect on the shape of the CD spectra. The compound (*P*)−**14a** displayed a strong negative Cotton effect from 250 to 300 nm with the maximum response at ≈255 nm, followed by a strong positive response at ≈370 nm, and a medium positive signal at ≈455 nm. In contrast, the intensity of the negative response at ≈250 nm of (*P*)−**15a** was decreased. The values of optical rotation of the free radicals **14a**–**c**, **15a**, and **15b** are significantly larger than that of their tertiary alcohol precursors **21a**–**c**, **22a**, and **22b** (**Table** **1**).

**Table 1:** Optical rotations.

Compound	14a	14b	14c	15a	15b
*P* isomer/${\left[\alpha \right]}_{D}^{20}$	−1330	−1050	−2290	−3330	−1400
*M* isomer/${\left[\alpha \right]}_{D}^{20}$	+1340	+1070	+2310	+3340	+1420
**Compound**	**21a**	**21b**	**21c**	**22a**	**22b**
*P* isomer/${\left[\alpha \right]}_{D}^{20}$	+265	+235	+233	+1166	+857
*M* isomer/${\left[\alpha \right]}_{D}^{20}$	−264	−235	−231	−1178	−861

### 2.4 X‐Ray Crystallographic Analyses and DFT Calculations

**Figure 7:**
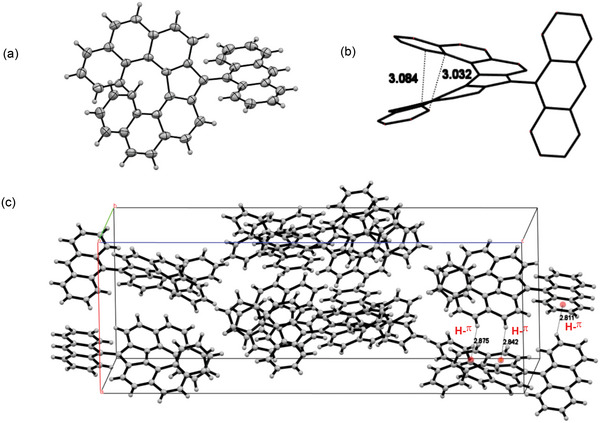
a) The ORTEP drawing of **14a** with thermal ellipsoids at 50% probability. b) The stick model of **14a**, and the shortest distances of carbon atoms were shown. c) The packing model of **14a**.

A single crystal of **14a** (CCDC 2071202) suitable for the X‐ray crystallographic analysis was successfully obtained by freezing its dry toluene solution at −20 °C (**Figure** **7**a). The crystal structure exhibited a monoradical that was well‐unassociated in the crystalline state. The nearest distance between two terminal aromatic rings was found to be 3.03 Å, which is within the twice of van der Walls radius of the carbon ≈3.4 Å (Figure 7b). This character indicates the possibility of delocalization of the radical via a through‐bond conjugation, as well as intramolecular through‐space conjugation. The packing model, depicted in Figure 7c, suggested the presence of intramolecular H–π interactions between neighboring skeletons.

**Figure 8:**
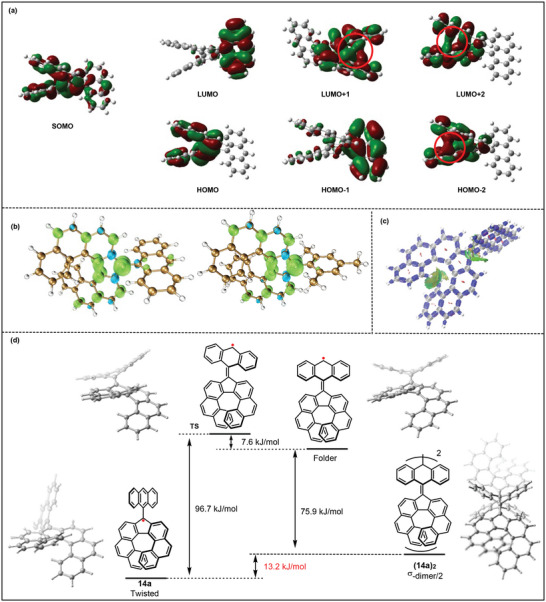
a) Selected molecular orbital distributions of **14a** from HOMO‐2 to LUMO+2, calculated at the B3LYP/6‐31+G (d, p) level. Red circles are introduced to highlight the through‐space conjugations. b) Spin density maps of **14a** (left) and **15a** (right) were calculated with the same calculation method. Green and blue surfaces represent α and β spin densities drawn at 0.004 e au^−3^ level, respectively. c) IRI analysis for the weak interaction of **14a**. d) Energy diagram for twisted **14a** to **(14a)_2_** (σ‐dimer) calculated at the B3LYP/6‐31+G (d, p) level.

To gain a deeper insight into the exceptional stability exhibited by **14a**, DFT calculations were performed (**Figure** **8**). The analysis revealed that the single electron presented in the [7]helical skeleton is well delocalized in the SOMO orbital. Additionally, through‐space conjugation effects were observed in multiple orbitals, including LUMO+1 (LUMO = lowest unoccupied molecular orbital), LUMO+2, and HOMO+2 (Figure 8a). Meanwhile, the orbital overlapping effect was also found in **15a** (see Supporting Information). The spin density maps obtained for **14a** and **15a** further provided evidence of the aforementioned unpaired electron delocalization, which was observed primarily in the fluorenyl moieties (Figure 8b). Additionally, IRI analysis exhibited a weak interaction between the upper and lower aromatic rings of the helical skeleton ideally, which was assigned to the through‐space interaction (Figure 8c). Importantly, we discovered that the ground state of **14a** must overcome a 96.7 kJ mol^−1^ energy barrier via **TS1** to assume a folded structure with the radical localized at the anthryl group (Figure 8d). From this folded radical form, it can undergo σ‐dimerization and transform into the dimer **(14a)_2_** (σ_2_). Intriguingly, **14a** was significantly more stable than its σ‐dimer **(14a)_2_** by DFT calculation, exhibiting a stability difference of 13.2 kJ mol^−1^, which was possible due to enlarged π‐conjugation system and the intramolecular through‐space conjugation of **14a**.

## 3 Discussion

**Figure 9:**
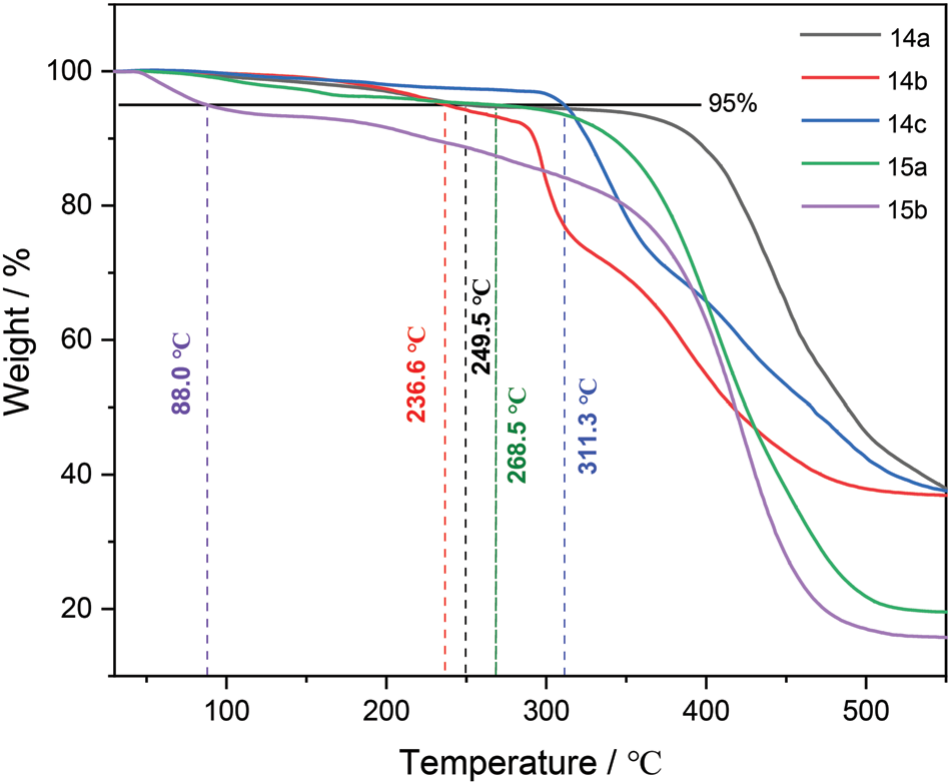
The TGA spectra of **14a–c** and **15a,b**.

The thermal stability of these radicals was analyzed and discussed using Thermogravimetric analysis (TGA) under a nitrogen atmosphere. As shown in **Figure** **9**, among the radicals tested, the temperature of 5% weight reduction of 9‐anthyl substituted radicals **14a–c** ranged from 249.5 to 311.3 °C, respectively. These results suggest that 9‐anthyl is an effective thermodynamic stabilization group. The studies also examined radicals with different substituents at the 10‐anthyl position, which revealed a moderate impact on thermal stabilities. Interestingly, radicals with polysubstituted phenyl as the steric shield group exhibited different thermal stability depending on *para*‐substitution. For instance, compound **15a** showed a temperature of 236.6 °C with 5% weight reduction, whereas **15b**, which had an *N, N*‐dimethylamino group at the *para* position of phenyl, demonstrated the lowest stability with a 5% weight reduction temperature of 88.0 °C. The lower stability of **15b** in TGA was consistent with the results obtained during the purification process, which showed gradual decomposition on silica gel column chromatography.

**Figure 10:**
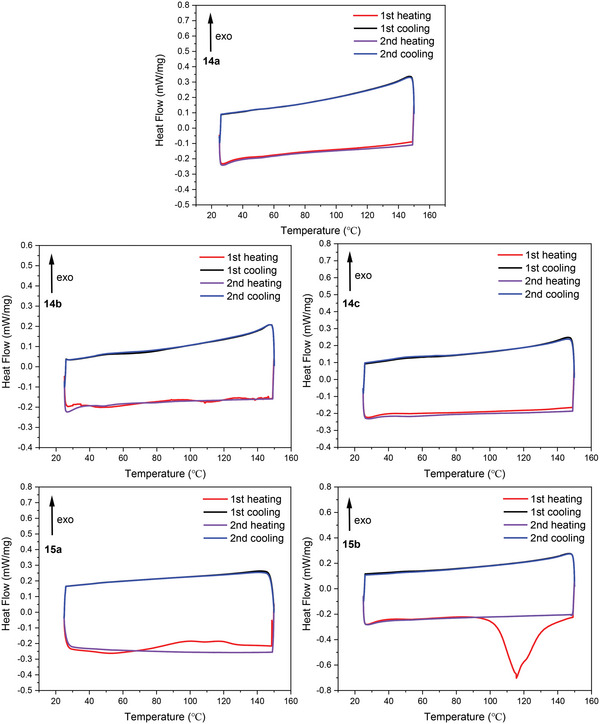
DSC curves of the corresponding stable radicals (under nitrogen flow).

Differential Scanning Calorimetry (DSC) analysis displayed that **14a**–**c** was fairly stable (**Figure** **10**). No clear absorption was observed upon heating, and the corresponding DSC curves for the first heating and the second heating were well matched. In contrast, broad endothermic curves were observed starting from 70 °C for compound **15a**, while a significant exotherm of **15b** was observed from ≈90 °C. All these results supported the conclusion that 9‐anthyl substituted radicals **14a–c** were more stable than the corresponding 2,6‐dimethylphenyl derivatives **15a**,**b**. The strong exotherm peak in the DSC curve of **15b** during the first heating was not observed again in the second heating cycle, indicating the decomposition of **15b** upon heating, which is consistent with the results obtained from the TGA spectra.

**Scheme 2:**
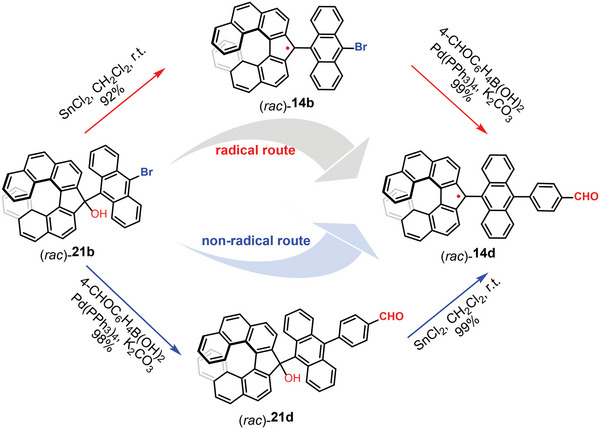
Radical and non‐radical routes for the synthesis of the functionalized radical **14d**.

The high stability of **14a** was further confirmed by HPLC analysis, which revealed no degradation after 28 days of stirring in hexadecane solution at room temperature under an air atmosphere (see Figure S3, Supporting Information). The stabilities of **14a**–**c** and **15a**,**b** in toluene in the air were analyzed by EPR upon the measurement of the intensity of the EPR signals at room temperature under dark conditions. Although there is a significant margin of error in measuring the strength of the EPR signals, no obvious decomposition of radicals **14a**–**c** can be found, while the half‐lives of **15a** and **15b** were estimated as 51 and 50 days, respectively (Figure S4, Supporting Information). Meanwhile, the transition‐metal catalyzed reaction offers a direct route for the modification of these radicals, with their unique radical structure remaining unaltered (**Scheme** **2**). For instance, a Pd‐catalyzed cross‐coupling reaction of (*rac*)−**14b** and 4‐formylphenylboronic acid achieved a high yield of radical (*rac*)−**14d**. To further confirm the structure of (*rac*)−**14d** obtained from the above cross‐coupling, an independent synthesis of (*rac*)−**14d** was conducted, namely Pd‐catalyzed Suzuki coupling, followed by the reduction of SnCl_2_. The radical compounds acquired through the two independent synthetic procedures were identical in EPR and UV–vis–NIR spectra, signifying the efficacy of both methodologies (see Figure S1, Supporting Information).

## 4 Conclusion

In conclusion, we have successfully synthesized a suite of highly stable carbon‐centered radicals that exhibit robustness to oxygen, acid, or base, and can be purified using column chromatography on silica gel. These radicals, bearing a [7]helical skeleton, were stabilized by through‐bond conjugation and intramolecular through‐space conjugation, evident in the molecular orbitals upon the DFT calculation. The steric shield capacity of the 9‐anthyl group was more effective in stabilizing the radical compared to the 2,6‐dimethylphenyl group. No dimerization of **14a** was observed regardless of in its solid state or solution, and the relative stabilities of **14a** and its σ‐dimers were calculated. Moreover, these stable radicals can be readily modified through a palladium‐catalyzed Suzuki coupling reaction, which provided a new avenue for radical fast functionalization. The chirality inherent in the helical structure also presents an opportunity to study their chiroptical properties and physiological activity.

## 5 Experimental Section

### Typical Procedure for the Preparation of Stable Carbon Radicals

An oven‐dried Schlenk tube was sequentially charged with corresponding cyclo‐fluorene *tert*‐alcohols (1.0 equiv.) and dry dichloromethane (0.02 m). SnCl_2_ (5.0 equivF.) was added and the mixture was stirred at room temperature for 1 h (otherwise stated). After full consumption of starting materials monitored by thin layer chromatography, water (10 mL) was added and the mixture was extracted with ethyl acetate (three times). The combined organic layer was washed with brine and dried over Na_2_SO_4_. The solvent was concentrated under reduced pressure and the residue was purified by column chromatography on silica gel to deliver the corresponding product, which was further characterized through EPR, UV–vis–NIR, and HRMS. Additionally, single isomers could be obtained via the reduction of optical *tert*‐alcohols (please see the Supporting Information for more details).

## Conflict of Interest

The authors declare no conflict of interest.

## Supporting information

Supporting Information

Supporting Information

## Data Availability

The data that support the findings of this study are available in the supplementary material of this article.
