# Supplementary material for: Conjugation‐Induced Spin Delocalization in Helical Chiral Carbon Radicals via Through‐Bond and Through‐Space Effects
Source: Adv Sci (Weinh). 2023 Oct 22;10(34):2304563. doi: 10.1002/advs.202304563 (PMC10700244; doi:10.1002/advs.202304563)
Supplement: Supplementary file 1 — Supporting Information [file ADVS-10-2304563-s001.pdf]

ADVANCED SCIENCE
Open Access

Supporting Information

for *Adv. Sci.*, DOI 10.1002/advs.202304563

Conjugation-Induced Spin Delocalization in Helical Chiral Carbon Radicals via Through-Bond and Through-Space Effects

Longhui Duan, Xiaoping Xue, Biqiong Hong and Zhenhua Gu**

1. Experimental section

1.1 General Information. All manipulations involving air- and/or moisture-sensitive compounds were carried out with the standard Schlenk technique under nitrogen in the oven or flame-dried glassware. Most of the reagents were used without further purification unless otherwise specified. Dichloromethane (CH_2Cl_2) was distilled from CaH_2 in a continuous still under an atmosphere of nitrogen. Tetrahydrofuran (THF) and diethyl ether (Et_2O) were distilled over sodium in the presence of benzophenone under an atmosphere of nitrogen. Flash column chromatography was performed using 200-300 mesh silica gel as the stationary phase. ^1H and ^{13}C NMR spectra were recorded on a Bruker AC-400 FT spectrometer using solvent residue as an internal reference (7.26 and 77.16 ppm for $CDCl_3$, 2.50 and 39.00 ppm for DMSO-d_6, respectively). Chemical shifts (δ) are reported in ppm, and coupling constants (J) are in Hertz (Hz). The following abbreviations were used to explain the multiplicities: s = singlet, d = doublet, t = triplet, q = quartet, m = multiplet, br = broad. High-resolution mass spectra (HRMS (ESI)) was recorded on a high-resolution mass spectrometer (WatersXEVO-G2Q-TOF). The cyclic voltammetry (CV) measurements were performed using a CH Instruments CHI660E electrochemical analyzer with a glass carbon disk as the working electrode, a platinum wire as the counter electrode, and Ag/Ag+ as the reference electrode. Sweep rates of 100 mV s^{-1} were utilized, and the ferrocenium/ferrocene redox couple was used as an external standard. EPR spectra were recorded on an electron paramagnetic resonance spectrometer (EPR) system at room temperature. UV-Vis-NIR absorption spectra were recorded on a UV−VIS-NIR scanning spectrophotometer. Circular dichroism (CD) spectra were recorded on a CD spectrometer system. Thermal stability measurements were performed in the temperature range of 30 – 800 °C on a thermogravimetric analyzer under nitrogen atmosphere at a heating rate of 10 K/min.

1.2 General Synthetic Procedures and Characterization Data

1.2.1 General Procedures for the Synthesis of *rac*-9*H*-Cyclopenta[1,2-c:4,3-c']diphenanthren-9-one (20)

4,5-Dibromo-9-fluorenone (17)

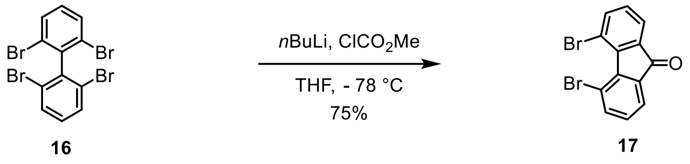

Under a nitrogen atmosphere, an oven-dried Schlenk tube was sequentially charged with 2,2',6,6'-tetrabromobiphenyl **16** (9.32 g, 20 mmol) and anhydrous THF (200 mL). After cooling down to -78 ℃, *n*BuLi (16.7 ml, 40 mmol, 2.4 M in THF) was added dropwise and stirred for 2 hours. Subsequently, methyl chloroformate (4.6 mL, 60 mmol) was added in one portion. The reaction was stirred for 1 h at -78 ℃ before being quenched by saturated aqueous NH_4Cl. The resulting yellow slush was extracted with Et_2O, washed with brine and the combined organic layer was dried over Na_2SO_4, filtered, and concentrated under reduced pressure. The residue was purified by column chromatography on silica gel (dichloromethane: hexanes = 1: 2) to deliver the corresponding product **17** as a yellow solid (5.10 g, 75% yield). ^1H NMR (500 MHz, $CDCl_3$) δ 7.79 – 7.68 (m, 4 H), 7.23 (t, J = 7.6 Hz, 2 H). The characterization data are consistent with previous reports.[1,2]

1,9-Bis{2-[(trimethlsilyl)ethynyl]phenyl}fluorenone (19)

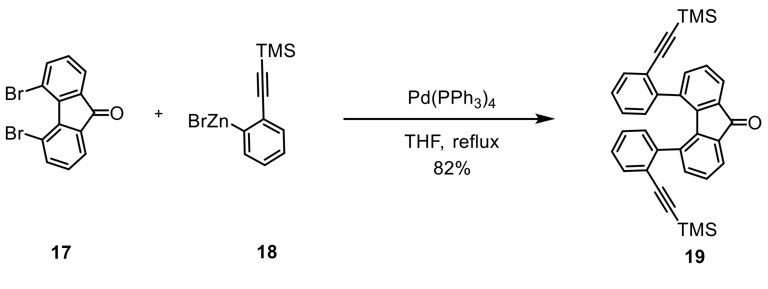

To a 200 mL oven-dry Schlenk flask was added 1-bromo-2-[(trimethylsilyl)ethynyl]-benzene (7.7 mL, 36 mmol, 3.0 equiv) and anhydrous THF (60 mL). The reaction was cooled down to -78 ℃ followed by the subsequent addition of *n*BuLi (15.0 mL, 36 mmol, 2.4 M in THF). After stirring for 40 min, the above lithium reagent was transferred to a solution of $ZnCl_2$ in THF at 0 ℃ via syringe (4.92 g anhydrous $ZnCl_2$ was dissolved in 60 mL THF) and stirred for 30 min to give the fresh prepared zinc reagent **18**. A solution of the fluorenone **17** (4.05 g, 12 mmol) and $Pd(PPh_3)_4$ (1.39 g, 1.2 mmol, 10 mol%) in THF was added to the solution. The resulting mixture was

warmed to room temperature and refluxed for 22 h. After the full consumption of starting materials monitored by TLC, saturated aqueous NH_4Cl was added and the solvent was concentrated under reduced pressure. The resulting residue was extracted with CH_2Cl_2 (three times), dried over Na_2SO_4. The solid was filtered and the filtrate was concentrated under reduced pressure. The residue was purified by column chromatography on silica gel (dichloromethane: hexanes =1: 4) to deliver a mixture of isomers **19** as a yellow solid (5.16 g, 82%). The obtained mixture of isomers was used without separation.

9*H*-Cyclopenta[1,2-c:4,3-c']diphenanthren-9-one (20)

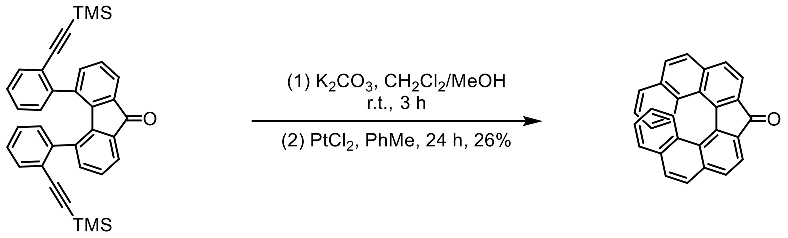

19 (*rac*)-**20**

To a 500 mL round-bottom flask was added 1,9-bis{2-[(trimethlsilyl)ethynyl]phenyl}fluorenone **19** (4.50 g, 8.6 mmol), K_2CO_3 (2.63 g, 19.0 mmol, 2.2 equiv), CH_2Cl_2 (20 mL) and MeOH (200 mL). The reaction was vigorously stirred at room temperature until completion of the reaction, which was monitored by TLC. After water (50 mL) was added, the resulting mixture was extracted with CH_2Cl_2. The organic layer was washed with brine, dried over Na_2SO_4, filtered, and the filtrate was concentrated under reduced pressure. The crude residue was purified by column chromatography on silica gel (dichloromethane: hexanes =1: 4) to deliver the corresponding product as a mixture of *cis*- and *trans*-isomers (~ 2.2:1) as a yellow solid.

A solution of the above alkyne and $PtCl_2$ (191.5 mg, 0.72 mmol) in toluene (200 mL) were refluxed for 18 h. After being cooled to room temperature, the reaction mixture was concentrated under reduced pressure, and the crude residue was purified by silica-gel column chromatography with chloroform as eluent to give the racemic **20** as a red solid (850.6 mg, 26% in two steps). ^1H NMR (400 MHz, $CDCl_3$) δ 7.90 (d, *J* = 7.6 Hz, 2 H), 7.75 (d, *J* = 7.5 Hz, 2 H), 7.68 – 7.57 (m, 4 H), 7.57 – 7.46 (m, 4 H), 7.22 – 7.03 (m, 2 H), 6.40 – 6.20 (m, 2 H).

The enantio-isomers of compound **20** were separated according to the previous report by preparative HPLC on a DICEL CHIEAPAK® IC-H column (eluent: hexane/CH_2Cl_2/Et_2NH = 75/25/0.1, flow rate = 1.0 mL/min, λ = 254 nm).[2]

1.2.2 General procedure for the synthesis of corresponding helical cyclo-fluorene *tert-*

alcohols

General procedure a:

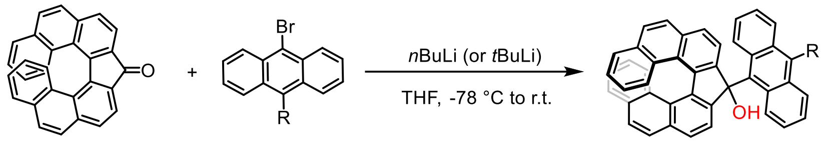

20 (R = H, Br, OMe) **21**

To an oven-dry Schlenk flask were added the corresponding bromides (0.60 mmol, 3.0 equiv) and anhydrous THF (or Et_2O) (5 mL). The mixture was cooled to -78 ℃ and *n*BuLi (0.23 mL, 0.66 mmol, 2.4 M in THF) was added dropwise. The reaction was stirred for 1 h, then a solution of (rac)-**20** (76.8 mg, 0.20 mmol, 1.0 equiv) in THF (5.0 mL) was added dropwise. After stirring for 1 h monitored by TLC, the resulting mixture was warmed to room temperature and saturated aqueous NH_4Cl was added. The mixture was extracted with CH_2Cl_2 (three times). The combined organic phase was washed with brine, dried over Na_2SO_4, filtrated and the filtrate was concentrated under reduced pressure. The residue was purified by column chromatography on silica gel (hexanes/ethyl acetate = 20:1) to deliver the corresponding product **21**.

General procedure b:

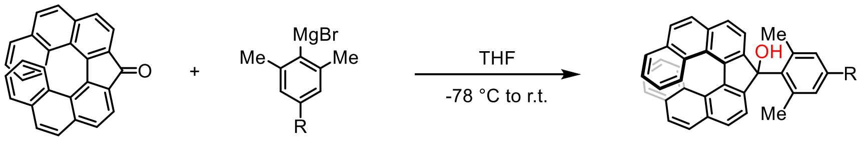

20 (R = Me or NMe_2) **22**

To a mixture of (*rac*)-**20** (76.8 mg, 0.20 mmol, 1.0 equiv) and anhydrous THF (5.0 mL) was added corresponding Grignard reagent (0.60 mmol, 3.0 equiv) dropwise at room temperature under stirring. The mixture was allowed to be stirred for 1 h monitored by TLC. After the full consumption of the starting material, saturated aqueous NH_4Cl was added. The reaction was extracted with CH_2Cl_2 (three times). The combined organic phase was washed with brine, dried over Na_2SO_4, filtrated and the filtrate was concentrated under reduced pressure. The residue was purified by column chromatography on silica gel (hexanes/ethyl acetate = 20:1) to deliver the corresponding product **22**.

9-(Anthracen-9-yl)-9*H*-cyclopenta[1,2-c:4,3-c']diphenanthren-9-ol (21a)

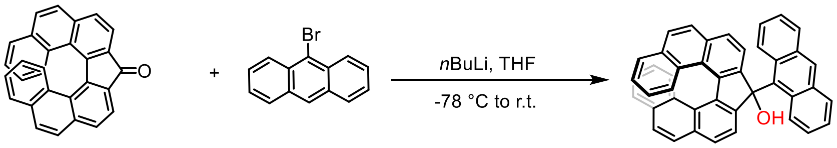

20 **S1** **21a**

(*rac*)-**21a** was prepared following **general procedure a**: The reaction of (*rac*)-**20** (76.8 mg, 0.20 mmol, 1.0 equiv), 9-bromoanthracene **S1** (154.2 mg, 0.60 mmol, 3.0 equiv) and *n*BuLi (0.23 mL, 0.66 mmol, 2.4 M in THF) in THF (5.0 mL) at -78 ℃ afforded (*rac*)-**21a** (107.20 mg, 96%) as a yellow solid (eluent for flash column chromatography: hexanes/ethyl acetate = 20:1). ^{1}H NMR (400 MHz, $CDCl_3$) δ 10.04 (d, *J* = 9.2 Hz, 1 H), 8.49 (s, 1 H), 8.10 (d, *J* = 8.4 Hz, 1 H), 8.00 (d, *J* = 7.8 Hz, 1 H), 7.88 (d, *J* = 8.4 Hz, 1 H), 7.82 – 7.75 (m, 3 H), 7.72 – 7.61 (m, 9 H), 7.58 – 7.52 (m, 1 H), 7.22 (d, *J* = 7.8 Hz, 1 H), 7.19 – 7.08 (m, 3 H), 6.72 – 6.65 (m, 1 H), 6.46 – 6.29 (m, 2 H), 2.88 (s, 1 H). ^{13}C NMR (101 MHz, $CDCl_3$) δ 156.0, 150.4, 137.8, 135.5, 134.0, 133.4, 133.0, 132.8, 132.4, 132.0, 131.9, 131.6, 131.3, 130.8, 130.0, 129.9, 129.8, 129.6, 128.93, 128.86, 128.8, 128.3, 127.7, 127.4, 127.3, 127.1, 127.02, 126.98, 126.68, 126.66, 126.2, 125.3, 124.8, 124.7, 124.4, 123.7, 123.6, 122.9, 121.7, 87.5. HRMS (ESI) calcd. for $C_{43}H_{26}ONa$ $[M+Na]^+$: 581.1881; Found: 581.1869.

(*P*)-**21a** was synthesized from (*P*)-**20** (19.2 mg, 0.05 mmol) in 94% yield (26.3 mg). $[\alpha]_D^{20}$ = -1330 (c 0.10, $CHCl_3$).

(*M*)-**21a** was synthesized from (*M*)-**20** (19.2 mg, 0.05 mmol) in 95% yield (26.5 mg). $[\alpha]_D^{20}$ = +1340 (c 0.10, $CHCl_3$).

9-(10-Bromoanthracen-9-yl)-9*H*-cyclopenta[1,2-c:4,3-c']diphenanthren-9-ol (21b)

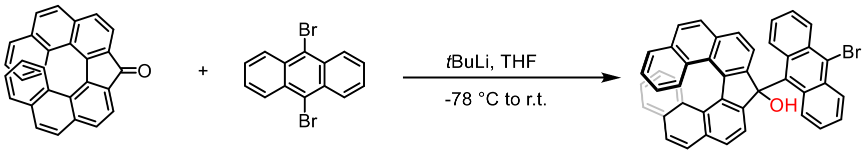

20 **S2** **21b**

(*rac*)-**21b** was prepared following **general procedure a:** The reaction of (*rac*)-**20** (76.8 mg, 0.20 mmol), 9,10-dibromoanthracene **S2** (201.6 mg, 0.60 mmol, 3.0 equiv) and *t*BuLi (1.15 mL, 1.50 mmol, 1.3 M in pentane) in THF (5.0 mL) at – 78 ℃ afforded (*rac*)-**21b** (91.8 mg, 72%) as a yellow solid (eluent for flash column chromatography: hexanes/ethyl acetate = 20:1). ^{1}H NMR (400 MHz, DMSO-d_6) δ 10.26 (d, *J* = 8.7 Hz, 1 H), 8.68 – 8.61 (m, 1 H), 8.41 (d, *J* = 8.3 Hz, 1 H), 8.02 – 7.89 (m, 2 H), 7.86 – 7.68 (m, 12 H), 7.35 – 7.28 (m, 1 H), 7.20 – 7.14 (m, 2 H), 7.11 (d, *J* = 7.8 Hz, 1 H), 6.92 (s, 1 H), 6.76 – 6.68 (m, 1 H), 6.43 – 6.32 (m, 2 H). ^{13}C NMR (101

MHz, DMSO-d_6) δ 155.8, 150.2, 137.0, 134.9, 134.1, 132.7, 132.6, 131.8, 131.0, 130.9, 130.3, 130.0, 129.7, 129.2, 129.02, 128.96, 128.1, 127.4, 127.1, 126.9, 126.8, 126.6, 126.5, 126.3, 126.2, 125.4, 125.2, 124.3, 123.9, 123.5, 123.1, 122.7, 122.0, 121.6, 85.6. HRMS (ESI) calcd. for $C_{43}H_{25}OBrNa$ [M+Na]$^+$: 659.0986; Found: 659.0973.

(*P*)-**21a** was synthesized from (*P*)-**20** (19.2 mg, 0.05 mmol) in 78% yield (24.9 mg). $[\alpha]_D^{20}$ = -1050 (c 0.10, $CHCl_3$).

(*P*)-**21a** was synthesized from (*P*)-**20** (19.2 mg, 0.05 mmol) in 82% yield (26.1 mg). $[\alpha]_D^{20}$ = +1070 (c 0.10, $CHCl_3$).

9-(10-Methoxyanthracen-9-yl)-9*H*-cyclopenta[1,2-c:4,3-c']diphenanthren-9-ol (21c)

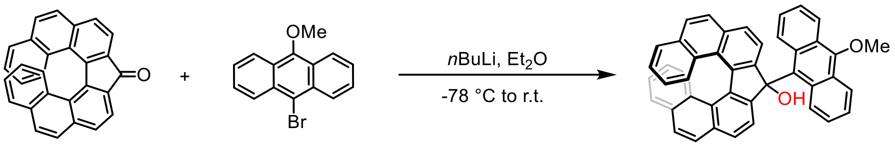

(*rac*)-**21c** was prepared following **general procedure a:** The reaction of (*rac*)-**20** (76.8 mg, 0.20 mmol), 9-bromo-10-methoxyanthracene **S3** (172.3 mg, 0.60 mmol, 3.0 equiv.) and *n*BuLi (0.23 mL, 0.66 mmol, 2.4 M in THF) in Et_2O (5.0 mL) afforded (*rac*)-**21c** (88.3 mg, 75%) as a yellow solid (eluent for flash column chromatography: hexanes/ethyl acetate = 20:1). ^{1}H NMR (500 MHz, $CDCl_3$) δ 10.09 (d, *J* = 9.2 Hz, 1 H), 8.70 – 8.46 (m, 1 H), 8.35 – 8.23 (m, 1 H), 8.05 (d, *J* = 7.8 Hz, 1 H), 7.89 – 7.78 (m, 3 H), 7.78 – 7.56 (m, 10 H), 7.38 – 7.26 (m, 1 H), 7.22 – 7.13 (m, 3 H), 6.78 – 6.64 (m, 1 H), 6.51 – 6.31 (m, 2 H), 4.21 (s, 3 H), 2.94 (s, 1 H). ^{13}C NMR (101 MHz, $CDCl_3$) δ 156.2, 153.6, 150.3, 137.7, 135.3, 133.9, 133.6, 133.3, 132.0, 131.8, 131.2, 130.7, 129.9, 129.8, 129.4, 129.2, 128.8, 128.4, 128.3, 127.7, 127.3, 127.3, 127.0, 127.00, 126.96, 126.7, 126.6, 126.5, 125.6, 125.3, 124.8, 124.7, 124.3, 123.63, 123.58, 123.0, 122.9, 122.5, 121.7, 87.3, 63.4. HRMS (ESI) calcd. for $C_{44}H_{28}O_2Na$ [M+Na]$^+$: 611.1987; Found: 611.2043.

(*P*)-**21c** was synthesized from (*P*)-**20** (19.2 mg, 0.05 mmol) in 85% yield (25.0 mg). $[\alpha]_D^{20}$ = -2290 (c 0.10, $CHCl_3$).

(*M*)-**21c** was synthesized from (*M*)-**20** (19.2 mg, 0.05 mmol) in 83% yield (24.4 mg). $[\alpha]_D^{20}$ = +2310 (c 0.10, $CHCl_3$).

9-Mesityl-9*H*-cyclopenta[1,2-c:4,3-c']diphenanthren-9-ol (22a)

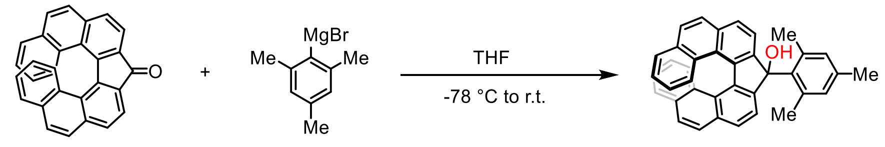

20 **S4** **22a**

(*rac*)-**22a** was prepared following **general procedure b:** The reaction of (*rac*)-**20** (76.8 mg, 0.20 mmol) and mesitylmagnesium bromide **S4** (0.60 mmol, 3.0 equiv) in THF (5.0 mL) at room temperature afforded (*rac*)-**22a** (98.1 mg, 98%) as a yellow solid (eluent for flash column chromatography: hexanes/ethyl acetate = 20:1). ^{1}H NMR (400 MHz, $CDCl_3$) δ 7.87 – 7.79 (m, 2 H), 7.77 – 7.65 (m, 6 H), 7.64 – 7.57 (m, 3 H), 7.55 (d, *J* = 8.2 Hz, 1 H), 7.20 – 7.03 (m, 3 H), 6.64 (s, 1 H), 6.37 – 6.26 (m, 2 H), 3.12 (s, 3 H), 2.38 (s, 1 H), 2.28 (s, 3 H), 1.48 (s, 3 H). ^{13}C NMR (101 MHz, $CDCl_3$) δ 153.2, 149.8, 139.7, 138.4, 136.5, 136.5, 136.2, 134.7, 133.7, 133.4, 132.8, 131.9, 131.7, 131.2, 131.1, 130.7, 129.71, 129.69, 128.6, 127.71, 127.69, 127.16, 127.14, 127.1, 127.0, 126.91, 126.86, 126.53, 126.50, 123.4, 122.3, 121.4, 86.3, 26.1, 21.7, 20.7. HRMS (ESI) calcd. for $C_{38}H_{28}ONa$ $[M+Na]^+$: 523.2038; Found: 523.2047.

(*P*)-**22a** was synthesized from (*P*)-**20** (19.2 mg, 0.05 mmol) in 96% yield (24.0 mg). $[\alpha]_D^{20}$ = -3330 (c 0.10, $CHCl_3$).

(*M*)-**22a** was synthesized from (*M*)-**20** (19.2 mg, 0.05 mmol) in 98% yield (24.5 mg). $[\alpha]_D^{20}$ = +3340 (c 0.10, $CHCl_3$).

9-[4-(Dimethylamino)-2,6-dimethylphenyl]-9*H*-cyclopenta[1,2-c:4,3-c']diphenanthren-9-ol (22b)

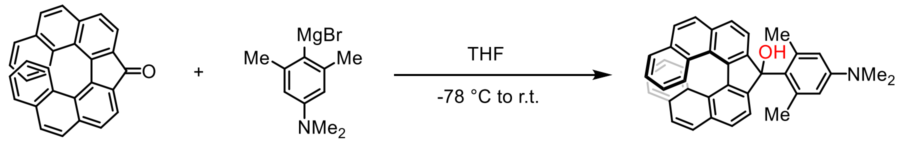

20 **S5** **22b**

(*rac*)-**22b** was prepared following **procedure b:** The reaction of (*rac*)-**20** (76.8 mg, 0.20 mmol), 4-NMe_2-2,6-Me_2-C_6H_2MgBr **S5** (0.60 mmol, 3.0 equiv) (fresh prepared from 4-(dimethylamino)-2,6-dimethylphenyl bromide and Mg turnings in THF at 70 ℃) in THF (5.0 mL) afforded (*rac*)-**22b** (96.4 mg, 91%) as a yellow solid (eluent for flash column chromatography: hexanes/ethyl acetate = 10:1 to 5:1). ^{1}H NMR (400 MHz, $CDCl_3$) δ 7.84 (d, *J* = 7.8 Hz, 1 H), 7.81 (d, *J* = 7.8 Hz, 1 H), 7.75 – 7.57 (m, 9 H), 7.55 (d, *J* = 8.3 Hz, 1 H), 7.14 – 7.07 (m, 2 H), 6.62 (d, *J* = 2.9 Hz, 1 H), 6.34 – 6.25 (m, 2 H), 6.20 (d, *J* = 2.9 Hz, 1 H), 3.12 (s, 3 H), 2.93 (s, 6 H), 2.35 (s, 1 H), 1.48 (s, 3 H). ^{13}C NMR (101 MHz, $CDCl_3$) δ 154.0, 150.2, 140.7, 138.2, 137.2, 136.3, 133.5,

133.3, 131.9, 131.7, 131.2, 130.7, 129.7, 129.64, 129.62, 128.6, 128.0, 127.69, 127.66, 127.2, 127.03, 126.99, 127.0, 126.83, 126.77, 126.7, 126.49, 126.46, 123.8, 123.4, 122.4, 121.5, 120.8, 86.2, 26.6, 22.3. HRMS (ESI) calcd. for $C_{39}H_{32}NO$ $[M+H]^+$: 530.2484; Found: 530.2481.

(*P*)-**22b** was synthesized from (*P*)-**20** (19.2 mg, 0.05 mmol) in 94% yield (24.8 mg). $[\alpha]_D^{20}$ = -1400 (c 0.10, $CHCl_3$).

(*M*)-**22b** was synthesized from (*M*)-**20** (19.2 mg, 0.05 mmol) in 94% yield (24.8 mg). $[\alpha]_D^{20}$ = +1420 (c 0.10, $CHCl_3$).

1.2.3 General procedure for the synthesis of corresponding helical radicals

Under nitrogen atmosphere, an oven-dried Schlenk tube was sequentially charged with corresponding cyclo-fluorene *tert*-alcohols (1.0 equiv) and dry dichloromethane (0.02 M). $SnCl_2$ (5.0 equiv) was added and the mixture was stirred at room temperature for 1 h (otherwise stated). After full consumption of starting materials monitored by TLC, water (10 mL) was added and the mixture was extracted with ethyl acetate (three times). The combined organic layer was washed with brine and dried over Na_2SO_4. The solvent was concentrated under reduced pressure and the residue was purified by column chromatography on silica gel to deliver the corresponding product.

Synthesis of 14a

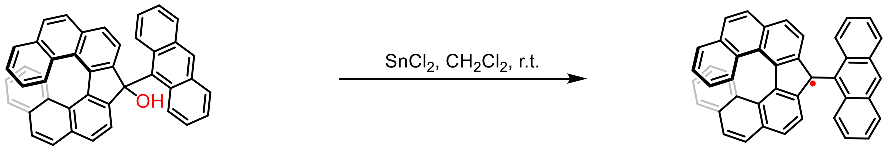

21a **14a**

The reaction of (*rac*)-**21a** (111.7 mg, 0.20 mmol, 1.0 equiv), $SnCl_2$ (189.6 mg, 1.00 mmol, 5.0 equiv) in CH_2Cl_2 (10 mL) at room temperature for 1 h afforded (*rac*)-**14a** (104.2 mg, 96%) as a deep green solid (eluent for flash column chromatography: hexanes/ethyl acetate/dichloromethane = 10:1:1).

UV−Vis-NIR (CH_2Cl_2, λ (nm)): 598 nm, 563 nm, 679 nm.

EPR: (toluene, T = 298 K) g = 1.9987.

HRMS (ESI^+) m/z: calcd for $C_{43}H_{26}$ $[M+H]^+$: 542.2029; Found: 542.1989.

(*P*)-**14a** was synthesized from (*P*)-**21a** (16.8 mg, 0.03 mmol) in 98% yield (15.9 mg). $[\alpha]_D^{20}$ = +265.2 (c 1×10^{-3}, $CHCl_3$).

(*M*)-**14a** was synthesized from (*M*)-**21a** (16.8 mg, 0.03 mmol) in 96% yield (15.6 mg). $[\alpha]_D^{20}$ = -263.8 (c 1×10^{-3}, $CHCl_3$).

Synthesis of 14b

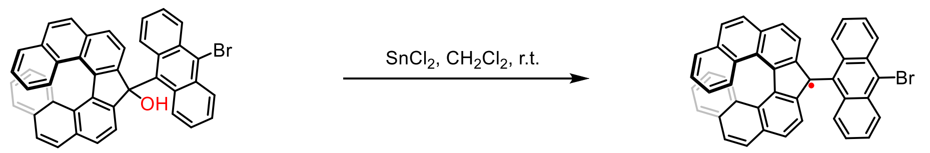

21b **14b**

The reaction of (*rac*)-**21b** (127.5 mg, 0.20 mmol, 1.0 equiv), $SnCl_2$ (189.6 mg, 1.00 mmol, 5.0 equiv) in CH_2Cl_2 (10 mL) at room temperature for 1 h afforded (*rac*)-**14b** (114.2 mg, 92%) as a deep green solid (eluent for flash column chromatography: hexanes/ethyl acetate/dichloromethane = 10:1:1).

UV−Vis-NIR (CH_2Cl_2, λ (nm)): 564 nm, 599 nm, 683 nm.

EPR: (toluene, T = 298 K) g = 1.9987.

HRMS (ESI$^+$) m/z: calcd for $C_{43}H_{25}Br$ $[M+H]^+$: 620.1134; Found: 620.1126.

(*P*)-**14b** was synthesized from (*P*)-**21b** (16.8 mg, 0.03 mmol) in 95% yield (18.2 mg) $[\alpha]_D^{20}$ = +235.2 (c 1×10^{-3}, $CHCl_3$).

(*M*)-**14b** was synthesized from (*M*)-**21b** (16.8 mg, 0.03 mmol) in 97% yield (18.6 mg). $[\alpha]_D^{20}$ = -234.6 (c 1×10^{-3}, $CHCl_3$).

Synthesis of 14c

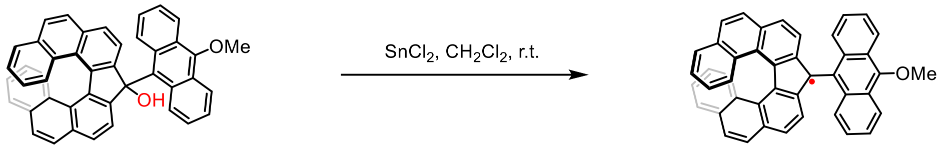

21c **14c**

The reaction of (*rac*)-**21c** (117.4 mg, 0.20 mmol, 1.0 equiv), $SnCl_2$ (189.6 mg, 1.00 mmol, 5.0 equiv) in CH_2Cl_2 (10 mL) at room temperature for 5 h afforded (*rac*)-**14c** (108.6 mg, 95%) as a deep green solid (eluent for flash column chromatography: hexanes/ethyl acetate/dichloromethane = 10:1:1).

UV−Vis-NIR (CH_2Cl_2, λ (nm)): 581 nm, 622 nm, 677 nm.

EPR: (toluene, T = 298 K) g = 1.9988.

HRMS (ESI$^+$) m/z: calcd. for $C_{44}H_{28}O$ $[M+H]^+$: 572.2135; Found: 572.2099.

(*P*)-**14c** was synthesized from (*P*)-**21c** (17.7 mg, 0.03 mmol) in 94% yield (16.1 mg). $[\alpha]_D^{20}$ = +232.6 (c 1×10^{-3}, $CHCl_3$).

(*M*)-**14c** was synthesized from (*M*)-**21c** (17.7 mg, 0.03 mmol) in 95% yield (16.3 mg). $[\alpha]_D^{20}$ = -230.8 (c 1×10^{-3}, $CHCl_3$).

Synthesis of 15a

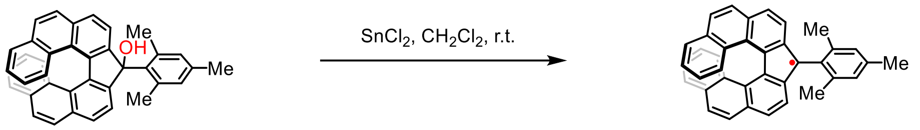

22a **15a**

The reaction of (rac)-**22a** (100.1 mg, 0.20 mmol, 1.0 equiv), $SnCl_2$ (189.6 mg, 1.00 mmol, 5.0 equiv) in CH_2Cl_2 (10 mL) at room temperature for 1 h afforded (rac)-**15a** (75.4 mg, 78%) as a black solid (eluent for flash column chromatography: hexanes/ethyl acetate/dichloromethane = 10:1:1 to dichloromethane).

UV−Vis-NIR (CH_2Cl_2, λ (nm)): 563 nm, 622 nm, 665 nm.

EPR: (toluene, T = 298 K) g = 1.9988.

HRMS (ESI$^+$) m/z: calcd. for $C_{38}H_{28}$ $[M+H]^+$: 484.2186; Found: 484.2152.

(*P*)-**15a** was synthesized from (*P*)-**22a** (15.0 mg, 0.03 mmol) in 76% yield (11.0 mg). $[\alpha]_D^{20}$ = +1165.8 (c 1×10^{-3}, $CHCl_3$).

(*M*)-**15a** was synthesized from (*M*)-**22a** (15.0 mg, 0.03 mmol) in 78% yield (11.3 mg). $[\alpha]_D^{20}$ = -1178.4 (c 1×10^{-3}, $CHCl_3$).

Synthesis of 15b

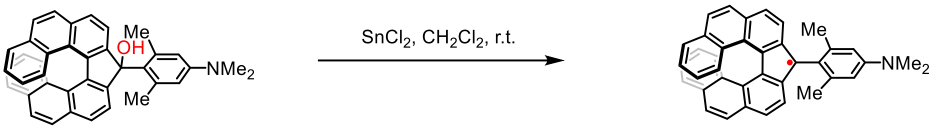

22b **15b**

The reaction of (*rac*)-**22b** (105.9 mg, 0.20 mmol, 1.0 equiv), $SnCl_2$ (189.6 mg, 1.00 mmol, 5.0 equiv) in CH_2Cl_2 (10 mL) at room temperature for 12 h afforded (*rac*)-**15b** (43.1 mg, 42%) as a deep green solid (eluent for flash column chromatography: hexanes/ethyl acetate/dichloromethane = 10:1:1 to dichloromethane. Quick column chromatography is necessary to avoid decomposition of **15b**).

UV−Vis-NIR (CH_2Cl_2, λ (nm)): 551 nm, 665 nm, 731 nm.

EPR: (toluene, T = 298 K) g = 1.9988.

HRMS (ESI+) m/z: calcd. for $C_{39}H_{31}N$ [M+H]+: 513.2451; Found: 513.2433.

(*P*)-**15b** was synthesized from (*P*)-**22b** (15.9 mg, 0.03 mmol) in 52% yield (8.0 mg). $[\alpha]_D^{20}$ = +856.8 (c 1×10-3, $CHCl_3$).

(*P*)-**15b** was synthesized from (*P*)-**22b** (15.9 mg, 0.03 mmol) in 48% yield (7.4 mg). $[\alpha]_D^{20}$ = -861.5 (c 1×10-3, $CHCl_3$).

1.2.4 Derivatizations

Radical route for the synthesis of 14d

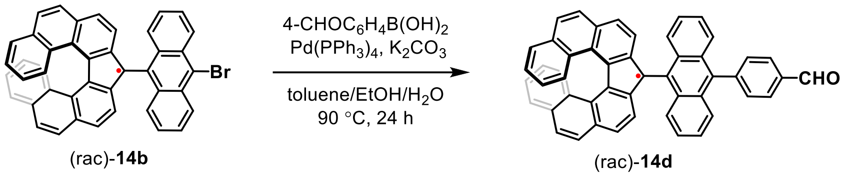

Under nitrogen atmosphere, an oven-dried seal tube was sequentially charged with (*rac*)-**14b** (124.1 mg, 0.20 mmol, 1.0 equiv), 4-CHO-$C_6H_4B(OH)_2$ (45.0 mg, 0.30 mmol, 1.5 equiv), $Pd(PPh_3)_4$ (4.6 mg, 2 mol%), K_2CO_3 (82.9 mg, 0.60 mmol, 3.0 equiv) in toluene/EtOH/H_2O (2.0 mL/0.5 mL/0.5 mL). The reaction was stirred at 90 ℃ for 24 h. After the reaction was completed monitored by TLC, it was cooled to room temperature and diluted with ethyl acetate, washed with water (three times) and brine, dried over anhydrous Na_2SO_4. The filtrate was concentrated under reduced pressure and the residue was purified by column chromatography on silica gel (hexanes/ethyl acetate/dichloromethane = 10:1:1) to afford (*rac*)-**14d** (127.8 mg, 99%).

UV−Vis-NIR (CH_2Cl_2, λ (nm)): 571 nm, 605 nm, 682 nm.

EPR: (toluene, T = 298 K) g = 2.0010.

HRMS (ESI+) m/z: calcd. for $C_{50}H_{30}O$ [M+H]+: 646.2291; Found: 646.2249.

Non-radical route for the synthesis of 14d

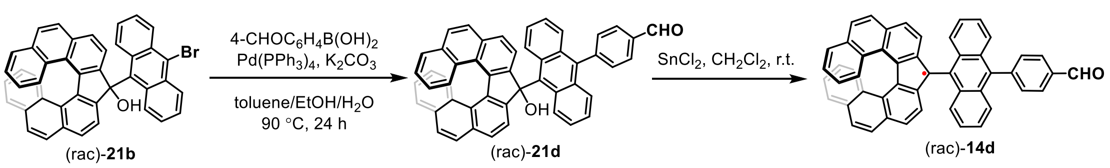

Under nitrogen atmosphere, an oven-dried seal tube was sequentially charged with (*rac*)-**21b** (127.5 mg, 0.20 mmol, 1.0 equiv), 4-CHO-$C_6H_4B(OH)_2$ (45.0 mg, 0.30 mmol, 1.5 equiv), $Pd(PPh_3)_4$ (4.6 mg, 2 mol%), K_2CO_3 (82.9 mg, 0.60 mmol, 3.0 equiv) in toluene/EtOH/H_2O (2.0 mL/0.5 mL/0.5 mL). The reaction was stirred at 90 ℃ for 24 h. After the reaction was completed monitored by TLC, it was cooled to room temperature and diluted with ethyl acetate, washed

with water (three times) and brine, then dried over anhydrous Na_2SO_4. The filtrate was concentrated under reduced pressure and the residue was purified by column chromatography on silica gel (ethyl acetate/hexanes = 1:20) to afford (*rac*)-**21d** as a yellow solid (129.9 mg, 98%). ^{1}H NMR (400 MHz, $CDCl_3$) δ 10.19 (s, 1 H), 10.12 (d, *J* = 9.8 Hz, 1 H), 8.18 – 8.07 (m, 3 H), 7.87 – 7.60 (m, 15 H), 7.49 – 7.38 (m, 2 H), 7.28 (d, *J* = 13.0 Hz, 1 H), 7.21 – 7.12 (m, 2 H), 7.05 – 6.99 (m, 1 H), 6.74 – 6.64 (m, 1 H), 6.44 – 6.35 (m, 2 H), 2.98 (s, 1 H). ^{13}C NMR (101 MHz, $CDCl_3$) δ 192.3, 156.1, 150.2, 146.6, 138.0, 137.6, 135.8, 135.4, 134.0, 133.5, 133.38, 132.41, 132.3, 132.02, 132.00, 131.9, 131.2, 131.1, 130.8, 130.02, 129.99, 129.9, 129.7, 129.1, 128.9, 128.44, 128.38, 127.7, 127.5, 127.4, 127.3, 127.1, 127.03, 127.00, 126.96, 126.8, 126.7, 126.3, 125.2, 124.9, 124.8, 124.3, 123.71, 123.66, 123.0, 121.7, 87.5. HRMS (ESI$^+$) m/z: calcd. for $C_{50}H_{31}O_2$ [M+H]$^+$: 663.2319; Found: 685.2308.

Under nitrogen atmosphere, an oven-dried Schlenk tube was sequentially charged with (*rac*)-**21d** (132.5 mg, 0.20 mmol, 1.0 equiv) and dry dichloromethane (10 mL). $SnCl_2$ (189.6 mg, 1.00 mmol, 5.0 equiv) was added and the mixture was stirred at room temperature. After full consumption of starting materials monitored by TLC, water (10 mL) was added and the mixture was extracted with ethyl acetate (three times). The combined organic layer was washed with brine and dried with Na_2SO_4. The solvent was concentrated under reduced pressure and the residue was purified by column chromatography on silica gel (hexanes/ethyl acetate/dichloromethane = 10:1:1 to dichloromethane) to deliver (*rac*)-**14d** (127.8 mg, 99%).

UV−Vis-NIR [CH_2Cl_2, λ (nm)]: 571 nm, 605 nm, 682 nm.

EPR: (toluene, T = 298 K) g = 2.0010.

HRMS (ESI$^+$) m/z: calcd. for $C_{50}H_{30}O$ [M+H]$^+$: 646.2291; Found: 646.2249.

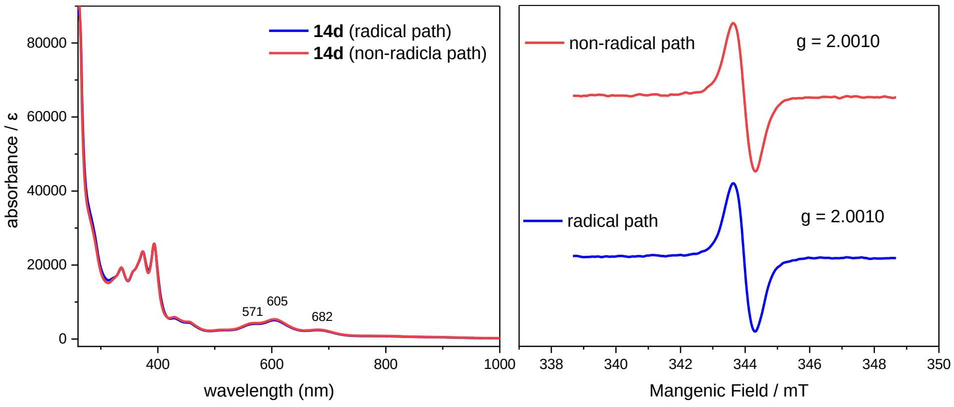

Figure S1. (Left) UV-vis-NIR spectra of **14d** from the radical pathway and non-radical pathway in Scheme 2 (CH_2Cl_2, c = 5×10^{-5} M, room temperature). (Right) EPR spectra of **14d** (toluene, c = 5×10^{-5} M, room temperature).

1.2.5 UV-Vis-IR spectrum

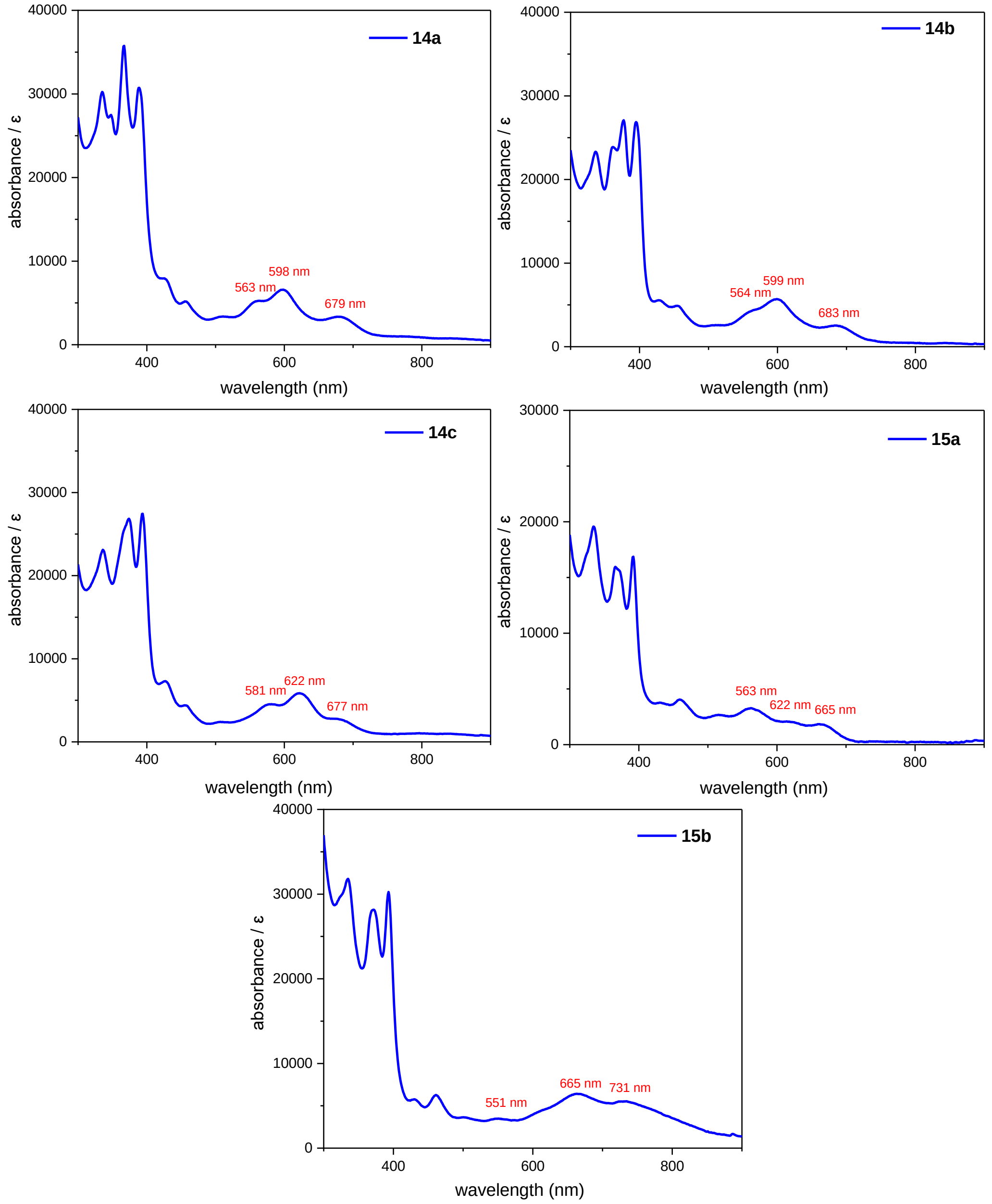

Figure S2. UV-vis-NIR spectra in CH_2Cl_2 of corresponding helical carbon radicals (c = 5×10^{-5} M, room temperature).

1.2.6 Time dependence of HPLC data for the investigation of stability of (*rac*)-14a.

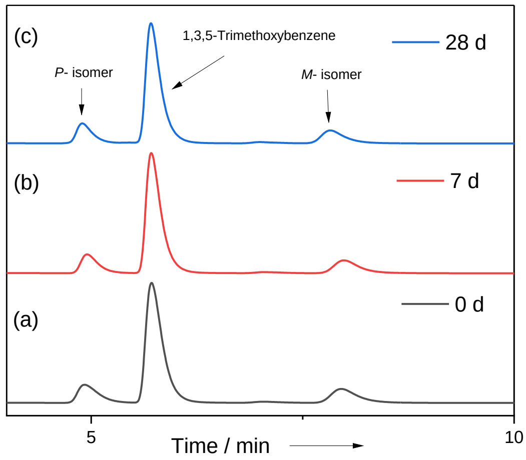

Figure S3. Time dependence of HPLC data for (*rac*)-**14a** tested in the air condition. (1,3,5-Trimethoxybenzene as internal standard. hexadecane solution, room temperature, AD-H, 98%, 1.0 mL/min, λ = 230).

1.2.7 Normalized intensities of time dependent ESR of corresponding radicals.

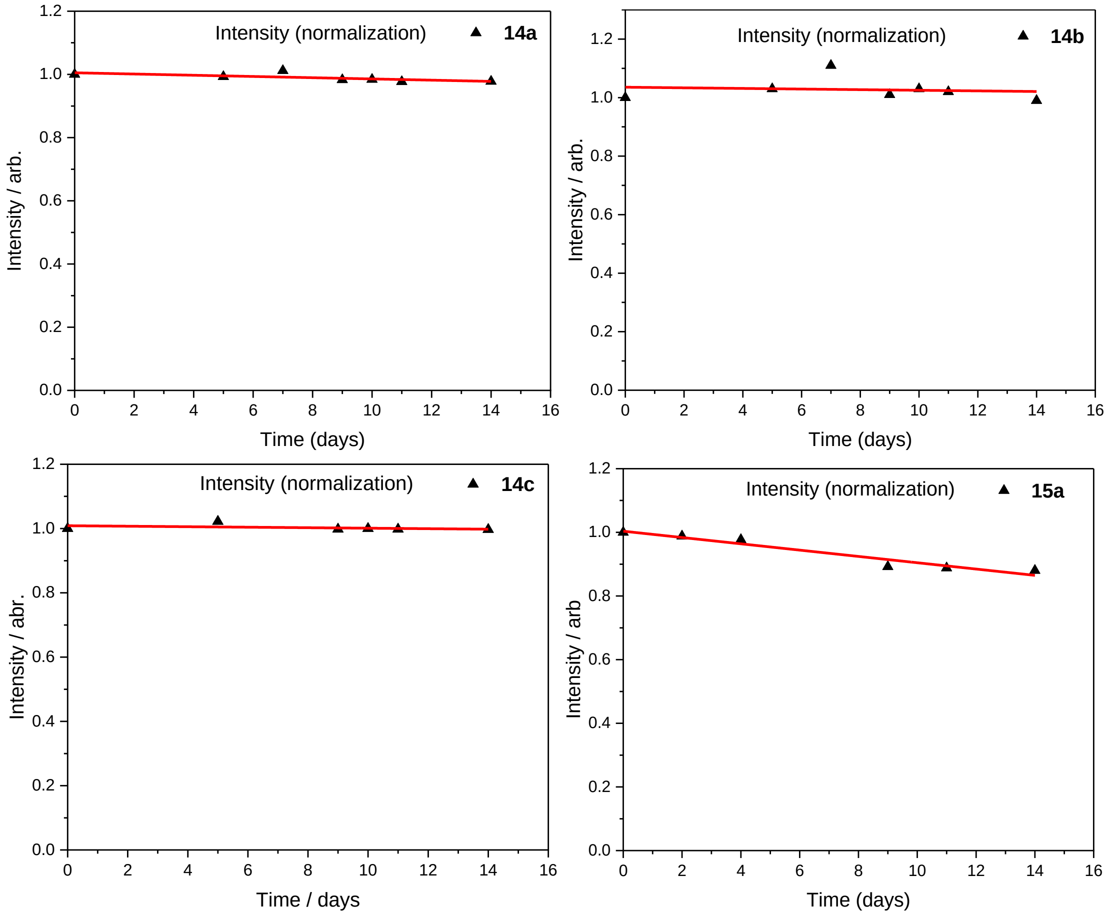

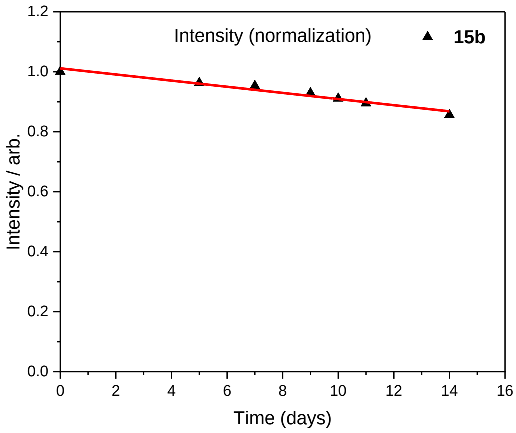

Figure S4. Normalized intensities of time dependent ESR (initial concentration is 5×10^{-4} M in toluene, at room temperature in air, g = 1.9988) measurements.

2. ^{1}H NMR and ^{13}C NMR Copies

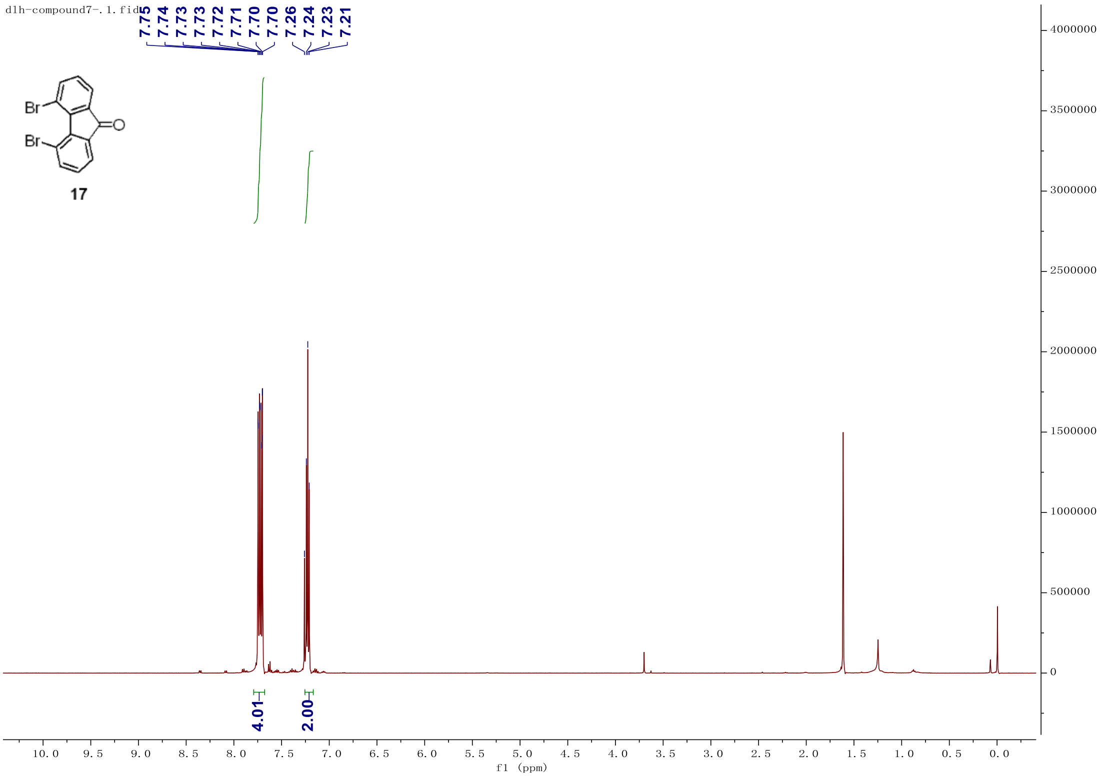

Figure S5. ^{1}H NMR spectra (500 MHz) of compound **17.**

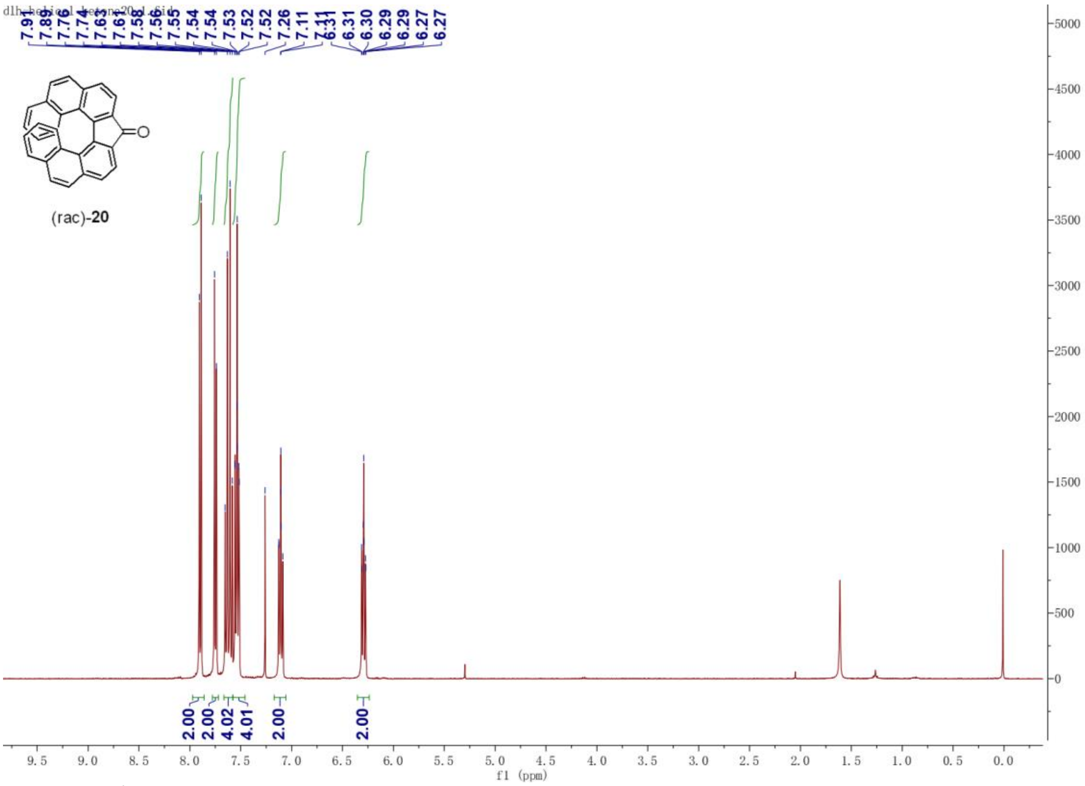

Figure S6. ^{1}H NMR spectra (400 MHz) of compound **20.**

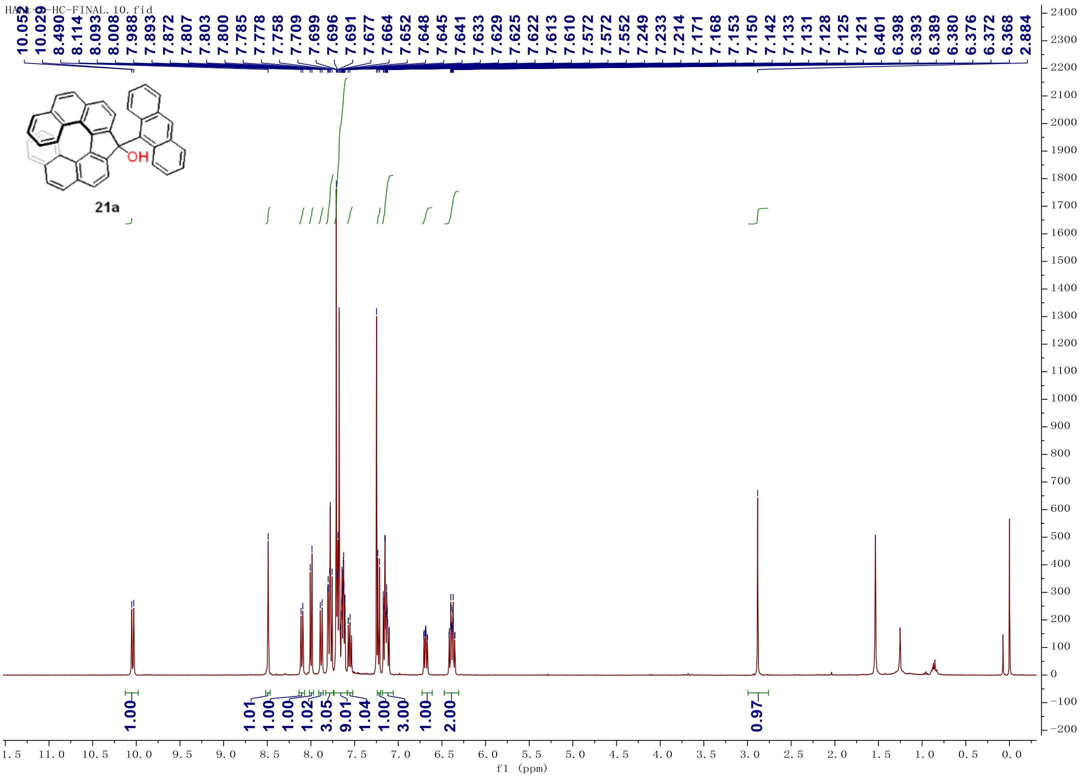

Figure S7. ^{1}H NMR spectra (400 MHz) of **21a.**

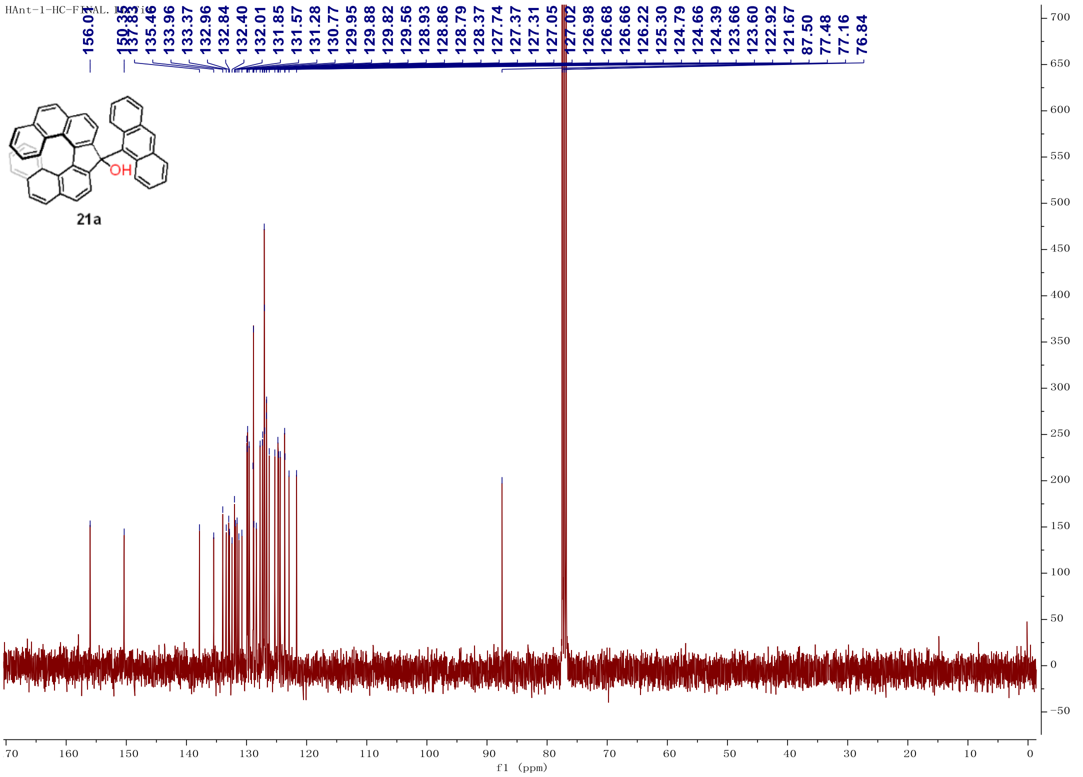

Figure S8. ^{13}C NMR spectra (101 MHz) of **21a.**

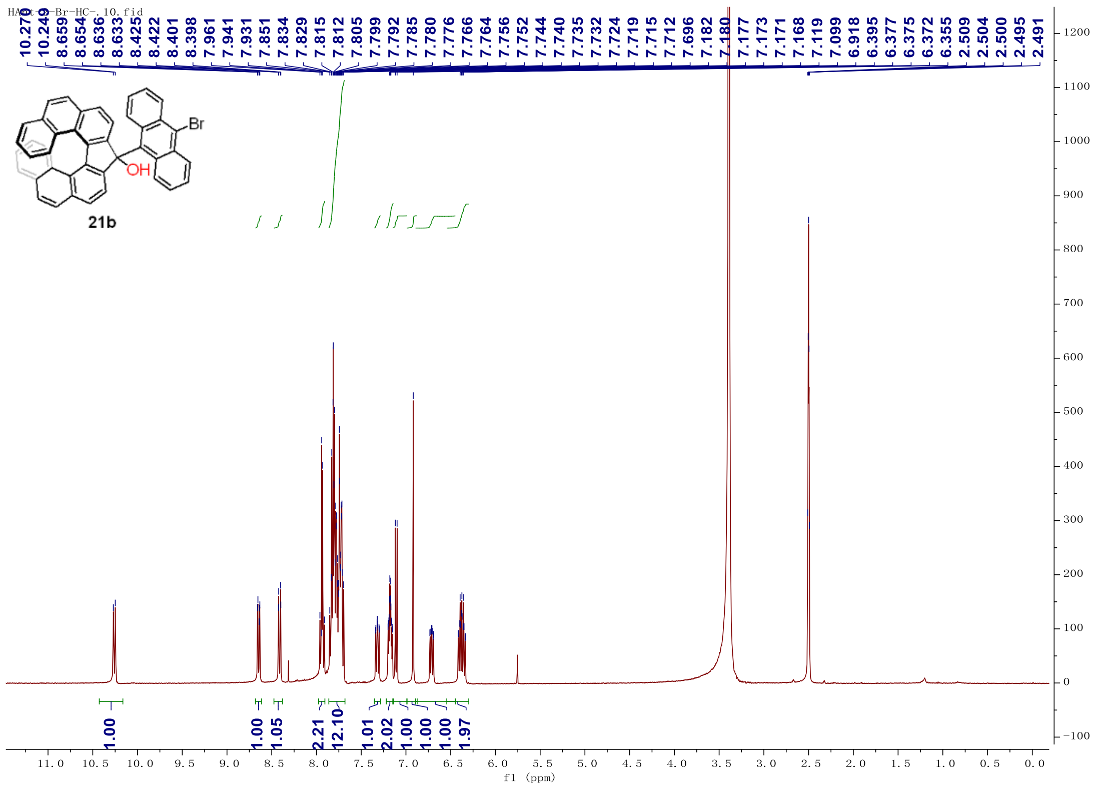

Figure S9. ^{1}H NMR spectra (400 MHz) of **21b.**

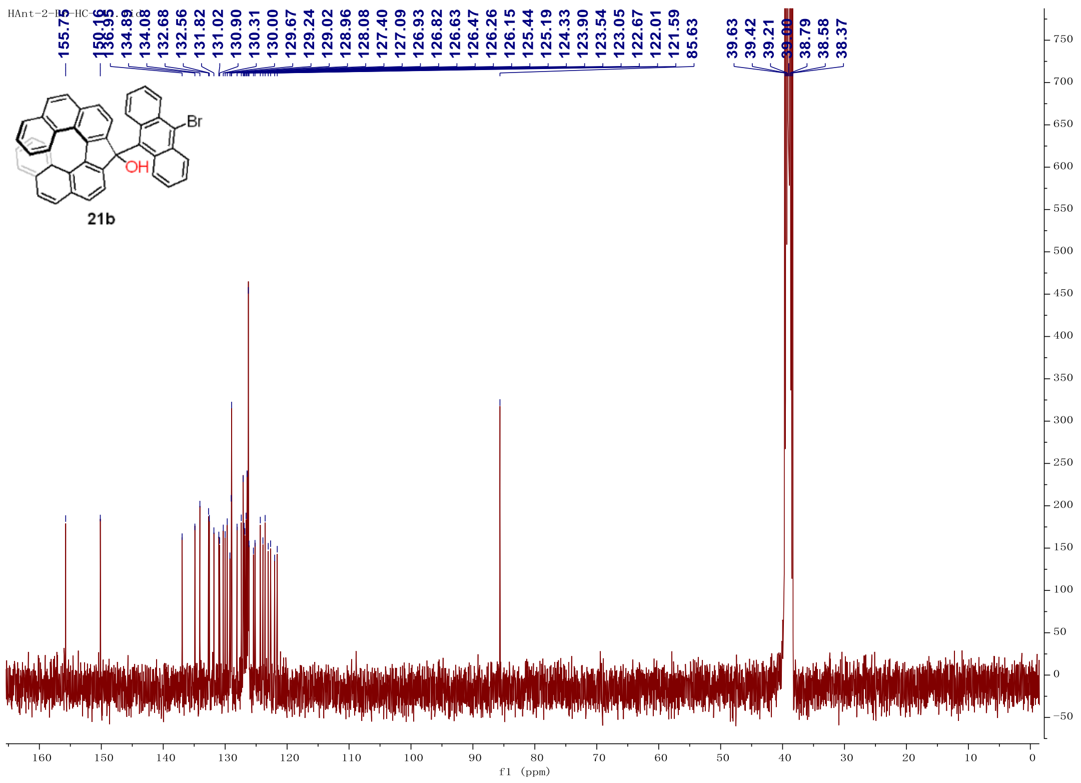

Figure S10. ^{13}C NMR spectra (101 MHz) of **21b.**

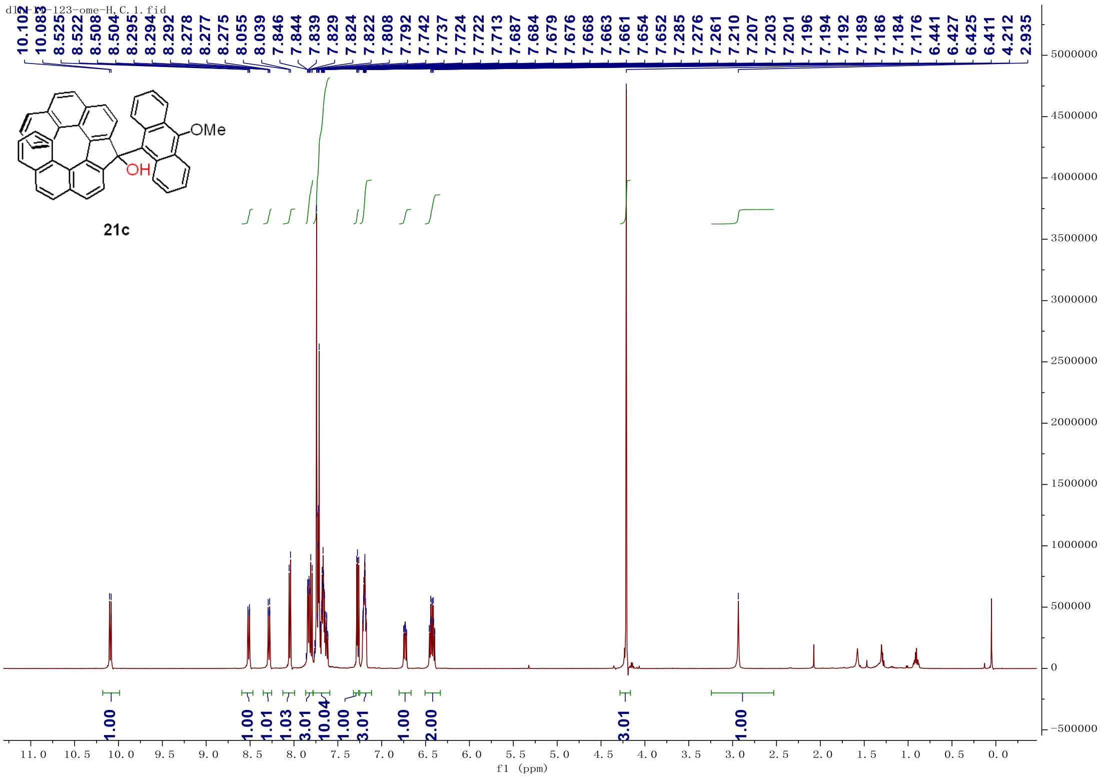

Figure S11. ^{1}H NMR spectra (500 MHz) of **21c.**

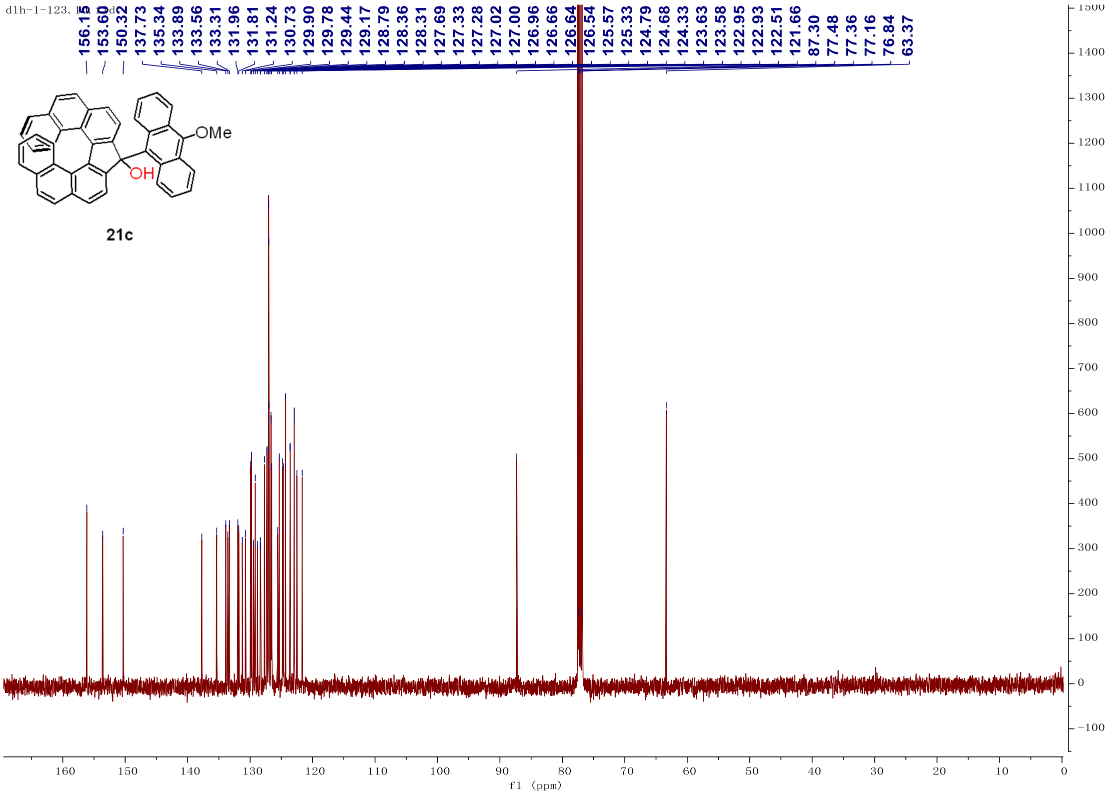

Figure S12. ^{13}C NMR spectra (101 MHz) of **21c.**

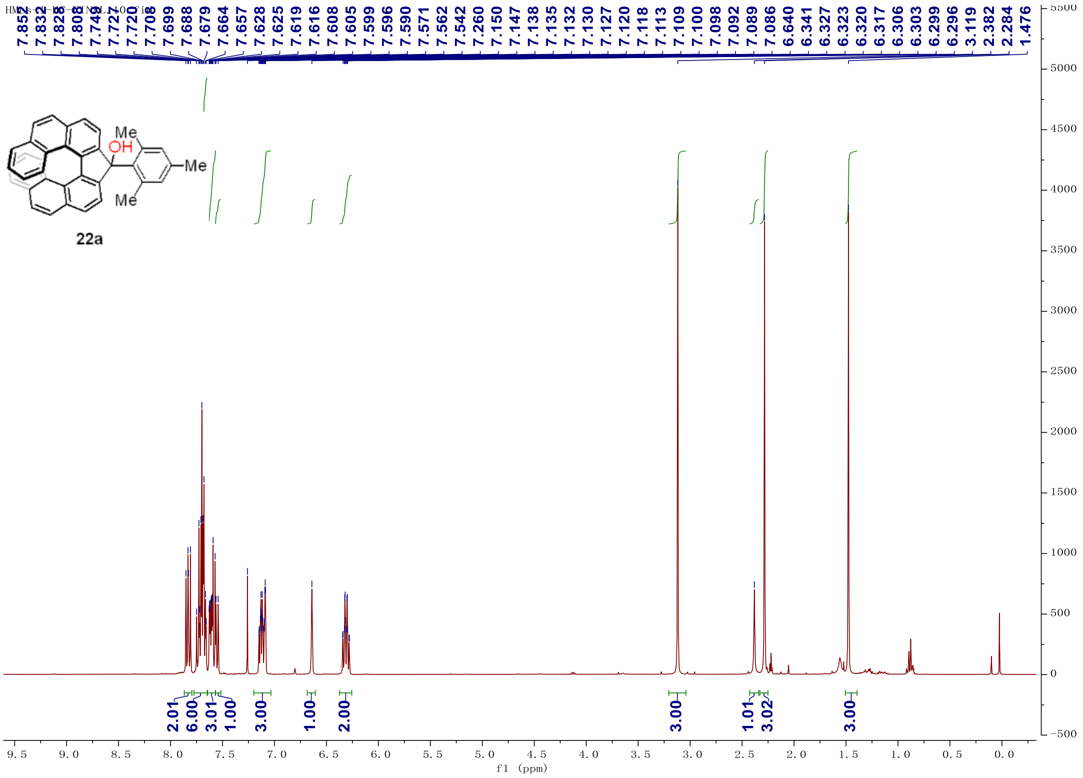

Figure S13. ^{1}H NMR spectra (400 MHz) of **22a.**

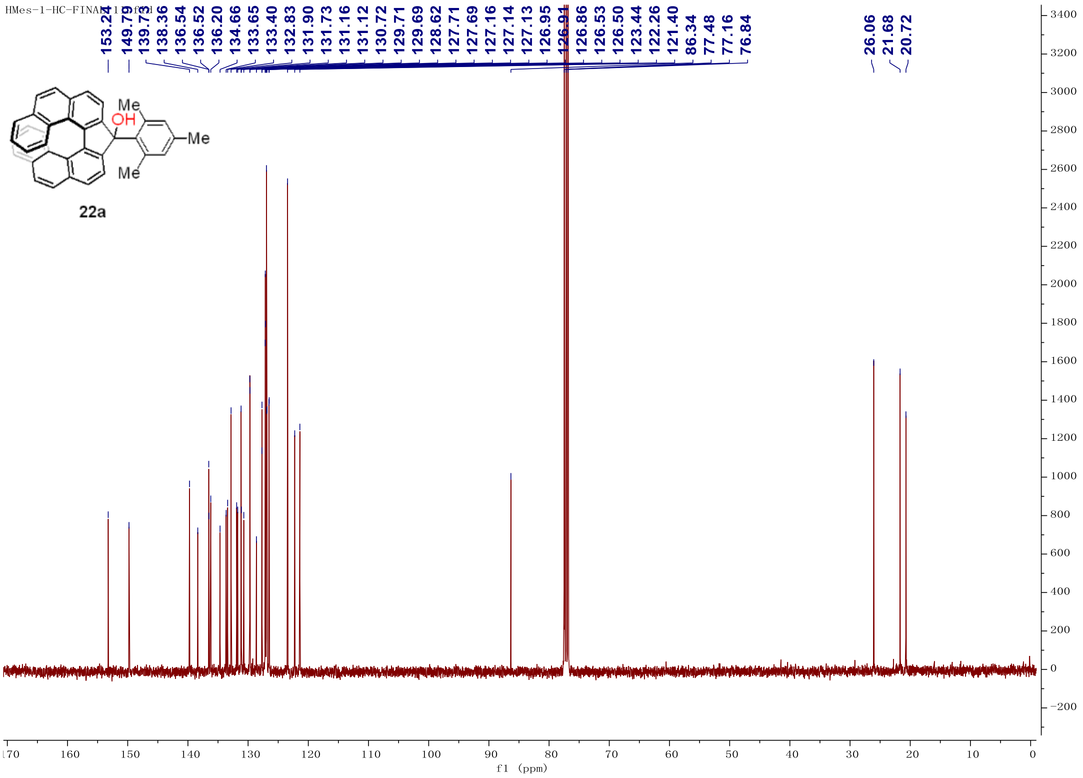

Figure S14. ^{13}C NMR spectra (101 MHz) of **22a.**

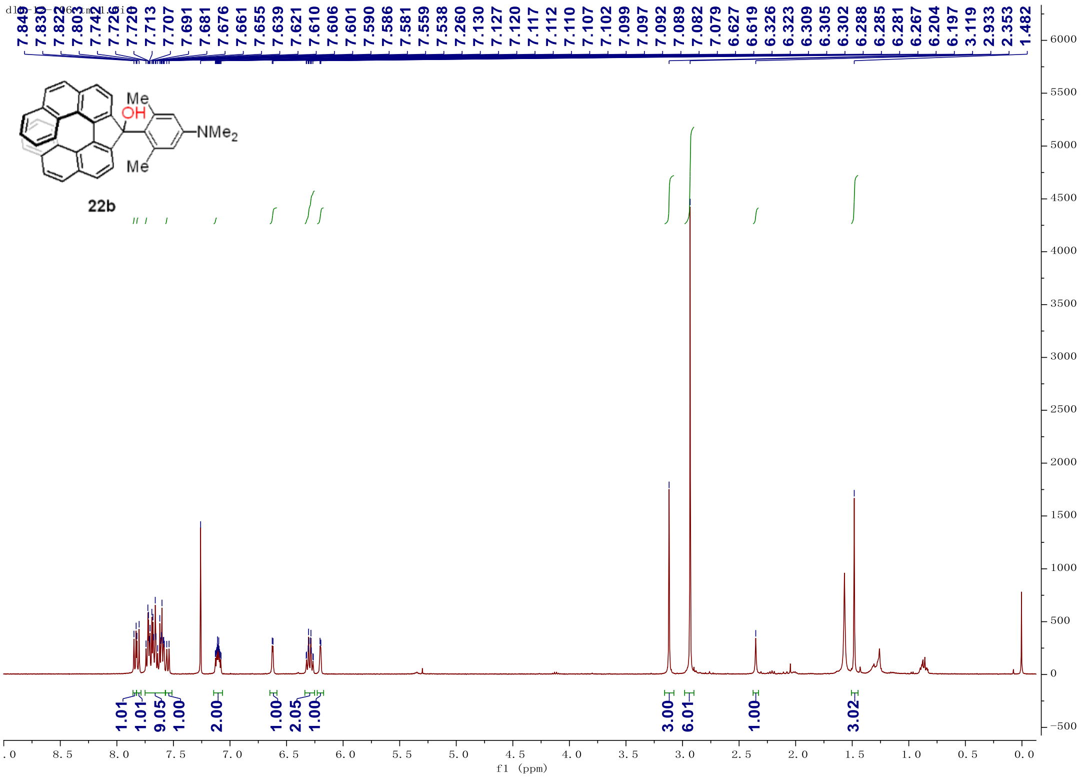

Figure S15. ^{1}H NMR spectra (400 MHz) of **22b.**

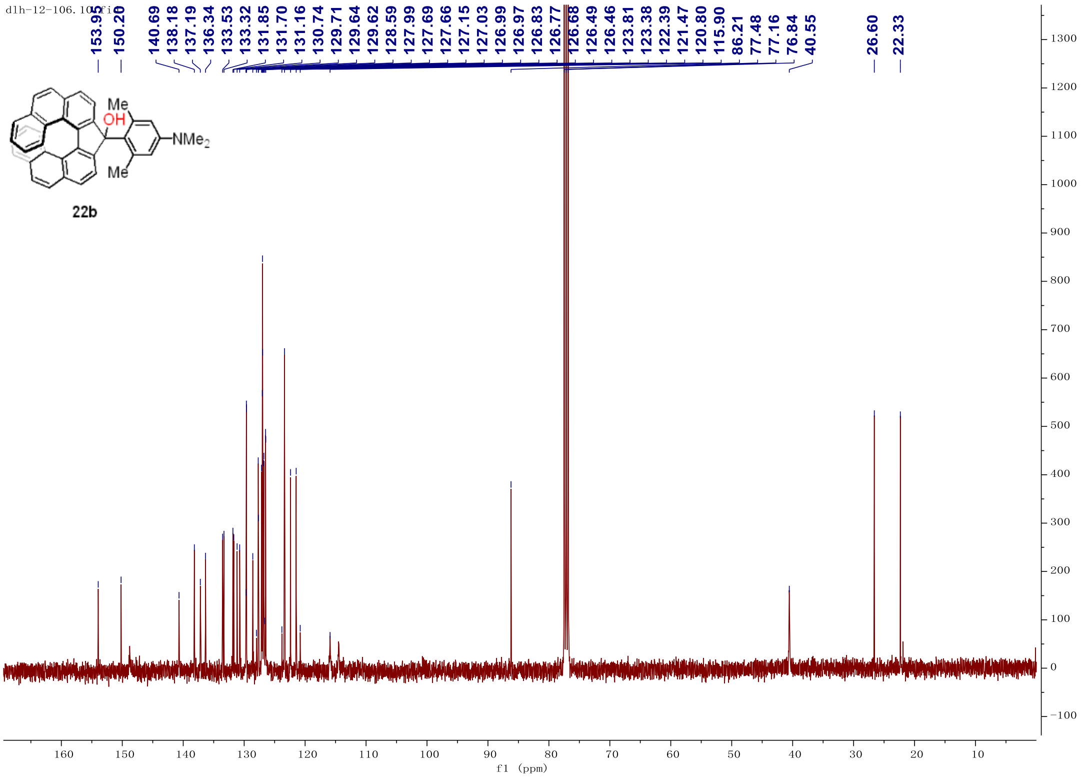

Figure S16. ^{13}C NMR spectra (101 MHz) of **22b.**

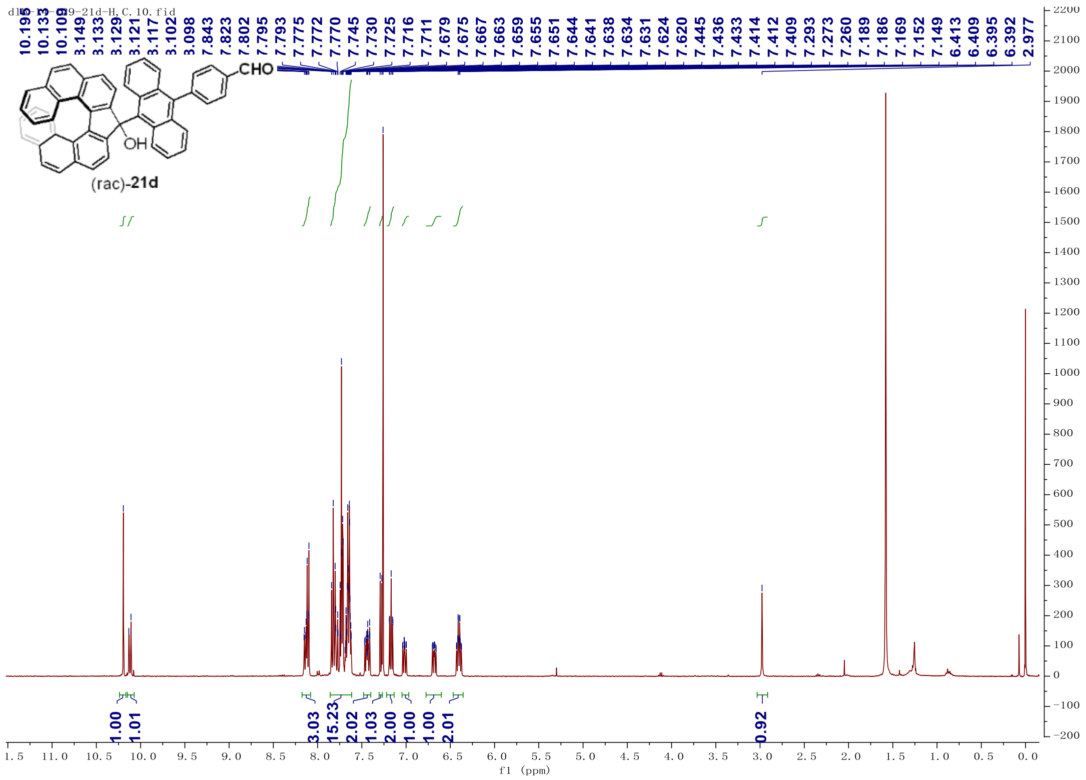

Figure S17. ^{1}H NMR spectra (400 MHz) of **21d.**

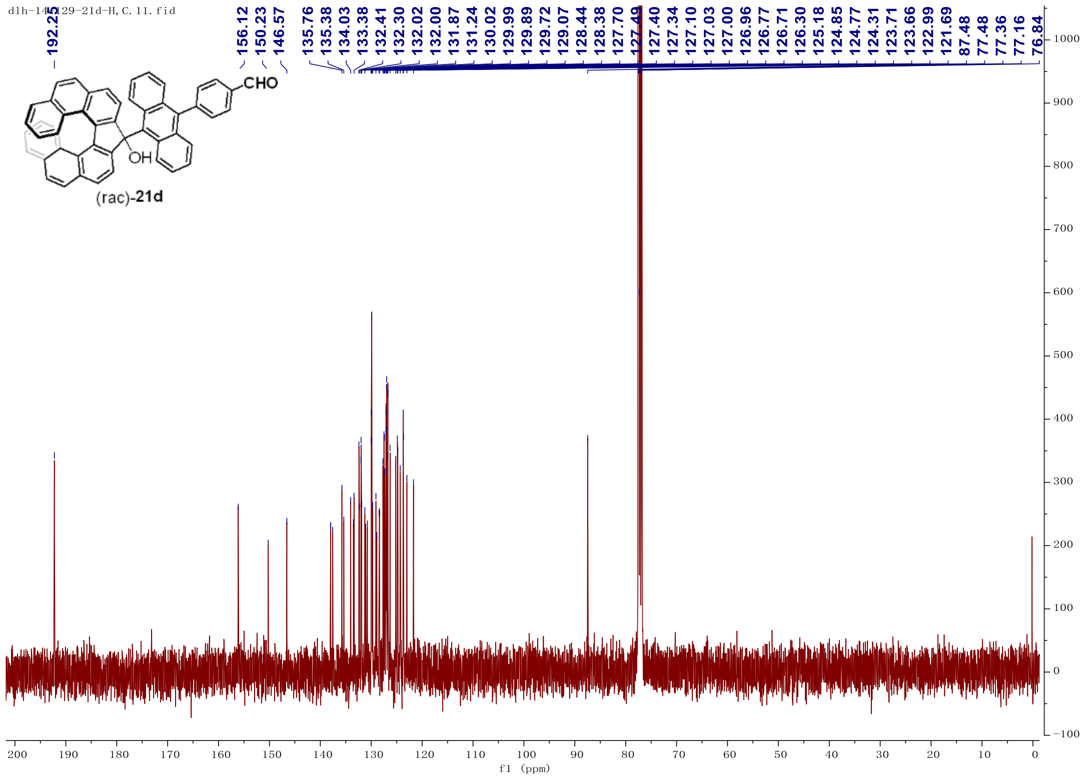

Figure S18. ^{13}C NMR spectra (101 MHz) of **21d.**

3. Crystal data for 14a

Table S1. Crystal data and structure refinement for **14a**.

Identification code	dlh-9
Empirical formula	$C_{43}H_{25}$
Formula weight	541.63
Temperature/K	179.99(10)
Crystal system	orthorhombic
Space group	$Pna2_1$
a/Å	12.1943(6)
b/Å	14.3939(7)
c/Å	33.639(2)
α/°	90
β/°	90
γ/°	90
Volume/Å^3	5904.5(6)
Z	8
ρ_{calc}g/cm^3	1.219
μ/mm^{-1}	0.526
F(000)	2264.0
Crystal size/mm^3	0.14 × 0.11 × 0.1
Radiation	Cu Kα (λ = 1.54184)
2Θ range for data collection/°	5.254 to 147.604
Index ranges	$-15 \le h \le 7$, $-14 \le k \le 17$, $-40 \le l \le 41$
Reflections collected	16739
Independent reflections	8625 [R_{int} = 0.0723, R_{sigma} = 0.0876]
Data/restraints/parameters	8625/1/775
Goodness-of-fit on F^2	0.975
Final R indexes [I>=2σ (I)]	R_1 = 0.0645, wR_2 = 0.1590
Final R indexes [all data]	R_1 = 0.0925, wR_2 = 0.1828
Largest diff. peak/hole / e Å^{-3}	0.39/-0.32
Flack parameter	0.5

Crystal structure determination of 14a

Crystal Data for $C_{43}H_{25}$ (M =541.63 g/mol): orthorhombic, space group $Pna2_1$ (no. 33), a = 12.1943(6) Å, b = 14.3939(7) Å, c = 33.639(2) Å, V = 5904.5(6) Å^3, Z = 8, T = 179.99(10) K, μ(Cu Kα) = 0.526 mm^{-1}, $Dcalc$ = 1.219 g/cm^3, 16739 reflections measured (5.254° ≤ 2Θ ≤ 147.604°), 8625 unique (R_{int} = 0.0723, R_{sigma} = 0.0876) which were used in all calculations. The final R_1 was 0.0645 (I > 2σ(I)) and wR_2 was 0.1828 (all data).

Table S2. Fractional Atomic Coordinates (×10^4) and Equivalent Isotropic Displacement Parameters (Å^2×10^3) for 14a. U_{eq} is defined as 1/3 of of the trace of the orthogonalised U_{IJ} tensor.

**Atom**	***x***	***y***	***z***	**U(eq)**
C1	5167(4)	5564(4)	5997.1(19)	35.5(13)
C2	4525(5)	4832(4)	6163(2)	43.4(14)
C3	4077(5)	4163(4)	5921(2)	46.0(15)
C4	4248(5)	4188(4)	5507(2)	43.9(14)
C5	4868(5)	4878(4)	5339(2)	41.3(14)
C6	5357(4)	5589(4)	5574.5(19)	35.7(13)
C7	5979(4)	6323(4)	5404.6(19)	35.3(12)
C8	6419(4)	7008(4)	5650(2)	38.8(13)
C9	6231(5)	6988(4)	6078.6(19)	37.7(13)
C10	5601(5)	6275(4)	6237(2)	41.7(14)
C11	6687(5)	7688(4)	6324(2)	46.1(14)
C12	7330(5)	8368(4)	6160(2)	48.6(16)
C13	7545(5)	8392(4)	5746(2)	48.5(17)
C14	7106(5)	7742(4)	5509(2)	43.5(14)
C15	6128(4)	6368(4)	4973.4(19)	34.7(12)
C16	5695(4)	7034(4)	4703.7(19)	33.4(12)
C17	6048(4)	6868(3)	4306.3(19)	32.1(12)
C18	6602(4)	5935(4)	4326(2)	34.3(12)
C19	6723(4)	5723(4)	4735.2(19)	35.8(12)
C20	7356(5)	4958(4)	4867(2)	42.5(14)
C21	7832(4)	4401(4)	4586(2)	41.2(14)
C22	7615(4)	4517(4)	4184(2)	40.0(15)
C23	6902(4)	5252(4)	4043(2)	36.4(13)
C24	6511(4)	5194(4)	3630.7(19)	35.4(12)
C25	7020(5)	4562(4)	3366(2)	45.1(15)
C26	7873(5)	3954(4)	3512(2)	48.0(16)
C27	8100(5)	3909(4)	3907(2)	43.3(15)
C28	6640(6)	4506(5)	2983(2)	56.9(17)
C29	5767(7)	5027(5)	2851(2)	59.2(18)
C30	5227(6)	5595(5)	3117(2)	51.1(16)
C31	5586(5)	5680(4)	3501.6(19)	36.5(13)
C32	4970(4)	7770(4)	4800(2)	39.0(13)
C33	4656(5)	8359(4)	4499.2(19)	37.9(13)
C34	5144(4)	8295(4)	4119.4(19)	34.2(12)
C35	4860(4)	8995(4)	3833(2)	38.9(13)
C36	5305(5)	8989(4)	3468(2)	41.0(14)

Table S2. Fractional Atomic Coordinates (×10^4) and Equivalent Isotropic Displacement Parameters (Å^2×10^3) for 14a. U_{eq} is defined as 1/3 of of the trace of the orthogonalised U_{IJ} tensor.

**Atom**	***x***	***y***	***z***	**U(eq)**
C37	6132(4)	8331(4)	3355.5(19)	37.0(13)
C38	6476(4)	7657(4)	3645.3(19)	34.9(12)
C39	5904(4)	7575(3)	4019.1(18)	30.3(11)
C40	7425(4)	7145(4)	3555(2)	35.8(13)
C41	7939(5)	7228(4)	3196(2)	45.8(15)
C42	7529(5)	7832(5)	2901(2)	47.8(15)
C43	6653(5)	8373(4)	2989(2)	43.9(14)
C44	5686(4)	278(4)	6578.9(19)	35.8(12)
C45	6604(4)	802(4)	6709(2)	37.8(12)
C46	6877(5)	845(4)	7104(2)	49.7(16)
C47	6232(6)	364(5)	7397(2)	57.5(18)
C48	5386(6)	-223(5)	7266(2)	53.6(17)
C49	5122(5)	-269(4)	6857(2)	44.7(14)
C50	4341(5)	-942(4)	6727(2)	45.3(14)
C51	4158(5)	-1079(4)	6340(2)	41.7(14)
C52	4681(4)	-511(4)	6039(2)	36.6(13)
C53	5370(4)	252(4)	6161.9(19)	34.0(12)
C54	5683(4)	892(4)	5864.5(18)	32.1(11)
C55	5557(4)	628(4)	5456.8(18)	32.4(12)
C56	4941(5)	-158(4)	5348(2)	38.8(13)
C57	4476(5)	-673(4)	5634(2)	41.4(14)
C58	6132(4)	1252(4)	5202.8(18)	33.1(12)
C59	6583(4)	1965(4)	5452.0(17)	32.2(11)
C60	7298(4)	2675(4)	5346(2)	37.2(12)
C61	7624(4)	3298(4)	5624(2)	38.8(13)
C62	7159(4)	3283(4)	6013.9(19)	34.7(12)
C63	6373(4)	2581(4)	6125.3(18)	32.6(11)
C64	6226(4)	1822(4)	5853.2(19)	31.7(11)
C65	5780(4)	2725(4)	6495.0(19)	33.4(12)
C66	6128(4)	3416(4)	6754.7(18)	34.5(12)
C67	6988(4)	4037(4)	6654(2)	41.4(14)
C68	7435(4)	3987(4)	6286(2)	39.8(14)
C69	5579(5)	3530(4)	7125(2)	42.2(14)
C70	4665(5)	2998(5)	7214(2)	48.2(15)
C71	4803(4)	2243(4)	6585(2)	37.6(13)
C72	4262(5)	2374(4)	6939(2)	42.4(13)

Table S2. Fractional Atomic Coordinates (×10[4]) and Equivalent Isotropic Displacement Parameters (Å[2]×10[3]) for 14a. U_{eq} is defined as 1/3 of of the trace of the orthogonalised U_{IJ} tensor.

**Atom**	***x***	***y***	***z***	**U(eq)**
C73	5108(5)	2645(4)	4692(2)	41.2(13)
C74	4668(5)	3307(4)	4443(2)	47.2(16)
C75	4876(5)	3319(4)	4039(2)	50.1(17)
C76	5507(5)	2645(5)	3872(2)	47.7(15)
C77	5976(4)	1924(4)	4107(2)	38.9(13)
C78	5788(4)	1928(4)	4525(2)	35.3(12)
C79	6254(4)	1214(4)	4767.3(18)	32.9(12)
C80	6858(4)	501(4)	4586.3(19)	32.4(11)
C81	7038(5)	501(4)	4170(2)	39.8(14)
C82	6591(5)	1211(4)	3940(2)	42.1(14)
C83	7367(4)	-221(4)	4814(2)	38.4(13)
C84	7988(5)	-891(4)	4635(2)	44.7(15)
C85	8134(5)	-895(4)	4223(2)	46.3(16)
C86	7697(5)	-225(4)	3994(2)	43.3(14)

Table S3. Anisotropic Displacement Parameters (Å[2]×10[3]) for **14a**. The Anisotropic displacement factor exponent takes the form: $-2\pi^2[h^2a^{*2}U_{11}+2hka^*b^*U_{12}+\ldots]$.

**Atom**	U_{11}	U_{22}	U_{33}	U_{23}	U_{13}	U_{12}
C1	29(2)	35(3)	43(4)	6(3)	-6(2)	0(2)
C2	40(3)	41(3)	49(4)	15(3)	5(3)	5(2)
C3	40(3)	36(3)	62(4)	9(3)	3(3)	-2(2)
C4	36(3)	38(3)	57(4)	-3(3)	3(3)	-4(2)
C5	38(3)	41(3)	45(4)	3(3)	-2(3)	0(2)
C6	31(2)	34(3)	42(3)	11(3)	-3(2)	-1(2)
C7	33(2)	32(3)	41(3)	0(3)	1(2)	-2(2)
C8	34(2)	35(3)	48(4)	4(3)	-7(3)	-1(2)
C9	35(3)	39(3)	39(3)	4(3)	-8(2)	1(2)
C10	39(3)	51(3)	36(3)	-4(3)	-1(3)	5(3)
C11	44(3)	47(3)	47(4)	-4(3)	-1(3)	2(3)
C12	44(3)	40(3)	61(5)	-15(3)	-9(3)	3(3)
C13	42(3)	38(3)	65(5)	7(3)	-11(3)	-7(3)
C14	42(3)	44(3)	44(4)	3(3)	-3(3)	-8(3)
C15	31(2)	32(3)	41(3)	6(2)	-1(2)	-7(2)
C16	33(2)	28(2)	40(3)	4(2)	0(2)	-4(2)
C17	28(2)	24(2)	44(3)	2(2)	3(2)	-0.1(19)

Table S3. Anisotropic Displacement Parameters ($Å^2×10^3$) for **14a**. The Anisotropic displacement factor exponent takes the form: $-2π^2[h^2a^{*2}U_{11}+2hka^*b^*U_{12}+…]$.

**Atom**	**U_{11}**	**U_{22}**	**U_{33}**	**U_{23}**	**U_{13}**	**U_{12}**
C18	26(2)	28(2)	48(4)	5(3)	0(2)	-1.7(19)
C19	32(2)	35(3)	40(3)	6(3)	1(2)	-3(2)
C20	41(3)	39(3)	47(4)	11(3)	-4(3)	-2(2)
C21	31(2)	32(3)	60(4)	10(3)	-4(3)	3(2)
C22	30(3)	32(3)	58(4)	2(3)	2(3)	1(2)
C23	29(2)	27(2)	53(4)	6(3)	7(2)	0(2)
C24	36(2)	29(2)	41(3)	5(2)	7(2)	-1(2)
C25	48(3)	36(3)	52(4)	-1(3)	10(3)	1(2)
C26	50(3)	33(3)	61(5)	1(3)	10(3)	8(2)
C27	37(3)	28(2)	65(4)	2(3)	3(3)	5(2)
C28	70(4)	55(4)	45(4)	-4(3)	9(4)	14(3)
C29	80(5)	61(4)	37(4)	-3(3)	1(4)	15(4)
C30	70(4)	43(3)	40(4)	7(3)	-3(3)	9(3)
C31	38(3)	32(3)	40(3)	6(3)	2(2)	0(2)
C32	36(3)	33(3)	48(4)	-1(3)	-1(3)	-1(2)
C33	37(3)	31(3)	45(4)	-8(3)	-1(3)	-1(2)
C34	32(2)	28(2)	43(3)	-5(2)	-5(2)	-2(2)
C35	31(2)	36(3)	50(4)	0(3)	-5(3)	7(2)
C36	40(3)	31(3)	52(4)	8(3)	-6(3)	2(2)
C37	34(2)	34(3)	44(3)	1(3)	0(2)	-2(2)
C38	37(2)	24(2)	44(3)	3(2)	0(2)	-4(2)
C39	30(2)	24(2)	37(3)	-5(2)	-3(2)	-3(2)
C40	29(2)	29(2)	49(4)	9(3)	1(2)	-1.3(19)
C41	35(3)	43(3)	59(4)	4(3)	9(3)	2(2)
C42	46(3)	53(3)	44(4)	10(3)	3(3)	-1(3)
C43	48(3)	42(3)	42(4)	9(3)	0(3)	-2(3)
C44	37(3)	32(3)	38(3)	0(2)	-2(2)	5(2)
C45	35(3)	36(3)	43(3)	3(3)	3(3)	0(2)
C46	47(3)	46(3)	56(4)	6(3)	-11(3)	-2(3)
C47	65(4)	66(4)	42(4)	-1(4)	-4(3)	-6(3)
C48	56(4)	62(4)	42(4)	12(3)	-3(3)	-9(3)
C49	48(3)	39(3)	48(4)	3(3)	3(3)	0(3)
C50	48(3)	40(3)	48(4)	10(3)	9(3)	-10(3)
C51	37(3)	33(3)	54(4)	2(3)	5(3)	-5(2)
C52	29(2)	28(2)	53(4)	-3(2)	3(3)	5(2)
C53	30(2)	30(2)	42(3)	-2(3)	1(2)	0(2)
C54	26(2)	33(3)	38(3)	2(2)	0(2)	0.2(19)

Table S3. Anisotropic Displacement Parameters (Å^2×10^3) for **14a**. The Anisotropic displacement factor exponent takes the form: $-2\pi^2[h^2a^{*2}U_{11}+2hka^*b^*U_{12}+\ldots]$.

**Atom**	**U_{11}**	**U_{22}**	**U_{33}**	**U_{23}**	**U_{13}**	**U_{12}**
C55	31(2)	31(2)	36(3)	-2(2)	1(2)	0(2)
C56	37(3)	33(3)	46(3)	-9(3)	1(3)	-3(2)
C57	39(3)	36(3)	49(4)	-9(3)	2(3)	-4(2)
C58	31(2)	33(3)	35(3)	0(2)	0(2)	6(2)
C59	30(2)	34(3)	33(3)	0(2)	-2(2)	5(2)
C60	37(3)	38(3)	37(3)	6(3)	-1(2)	-1(2)
C61	32(2)	35(3)	50(4)	4(3)	1(3)	-6(2)
C62	27(2)	28(2)	49(4)	3(2)	-2(2)	1(2)
C63	29(2)	31(2)	38(3)	-4(2)	-4(2)	0(2)
C64	25(2)	30(2)	41(3)	3(2)	-1(2)	4.0(19)
C65	29(2)	31(3)	40(3)	4(2)	-4(2)	1(2)
C66	38(3)	31(2)	34(3)	0(2)	-5(2)	5(2)
C67	33(3)	38(3)	53(4)	-5(3)	-6(3)	-4(2)
C68	34(3)	27(2)	58(4)	2(3)	-6(3)	-3(2)
C69	45(3)	40(3)	42(4)	-11(3)	-7(3)	6(2)
C70	49(3)	50(3)	46(4)	-11(3)	1(3)	10(3)
C71	33(2)	38(3)	42(3)	-4(3)	-3(2)	4(2)
C72	38(3)	42(3)	47(4)	-2(3)	2(3)	0(2)
C73	42(3)	34(3)	47(4)	-2(3)	-5(3)	6(2)
C74	40(3)	33(3)	69(5)	-1(3)	-10(3)	9(2)
C75	42(3)	41(3)	68(5)	17(3)	-7(3)	3(2)
C76	44(3)	52(4)	47(4)	14(3)	-5(3)	-9(3)
C77	31(2)	40(3)	47(4)	4(3)	2(3)	-9(2)
C78	31(2)	32(3)	43(3)	1(3)	-2(3)	3(2)
C79	32(2)	32(3)	34(3)	1(2)	-1(2)	-2(2)
C80	27(2)	32(2)	39(3)	-1(2)	0(2)	-2(2)
C81	33(3)	35(3)	51(4)	-6(3)	3(3)	-4(2)
C82	35(3)	49(3)	42(4)	-1(3)	2(3)	-2(2)
C83	34(3)	36(3)	45(4)	3(3)	1(3)	5(2)
C84	37(3)	35(3)	62(5)	0(3)	8(3)	6(2)
C85	37(3)	34(3)	68(5)	-11(3)	13(3)	0(2)
C86	44(3)	43(3)	43(4)	-6(3)	7(3)	-3(3)

Table S4. Bond Lengths for **14a.**

Atom	Atom	Length/Å	Atom	Atom	Length/Å
C1	C2	1.426(8)	C44	C45	1.418(8)
C1	C6	1.441(9)	C44	C49	1.405(9)
C1	C10	1.407(9)	C44	C53	1.455(9)
C2	C3	1.374(10)	C45	C46	1.369(10)
C3	C4	1.408(10)	C46	C47	1.438(10)
C4	C5	1.370(9)	C47	C48	1.405(10)
C5	C6	1.426(9)	C48	C49	1.414(10)
C6	C7	1.421(8)	C49	C50	1.426(9)
C7	C8	1.393(8)	C50	C51	1.335(9)
C7	C15	1.463(8)	C51	C52	1.451(8)
C8	C9	1.461(9)	C52	C53	1.443(8)
C8	C14	1.429(8)	C52	C57	1.405(9)
C9	C10	1.388(9)	C53	C54	1.413(8)
C9	C11	1.416(9)	C54	C55	1.431(8)
C11	C12	1.369(10)	C54	C64	1.494(7)
C12	C13	1.416(11)	C55	C56	1.407(8)
C13	C14	1.343(9)	C55	C58	1.424(8)
C15	C16	1.422(8)	C56	C57	1.341(9)
C15	C19	1.425(8)	C58	C59	1.434(8)
C16	C17	1.425(9)	C58	C79	1.474(8)
C16	C32	1.417(8)	C59	C60	1.391(8)
C17	C18	1.505(7)	C59	C64	1.433(8)
C17	C39	1.414(8)	C60	C61	1.352(9)
C18	C19	1.418(9)	C61	C62	1.430(9)
C18	C23	1.416(8)	C62	C63	1.442(7)
C19	C20	1.416(8)	C62	C68	1.406(9)
C20	C21	1.368(10)	C63	C64	1.436(8)
C21	C22	1.387(10)	C63	C65	1.454(8)
C22	C23	1.449(8)	C65	C66	1.390(8)
C22	C27	1.409(9)	C65	C71	1.411(8)
C23	C24	1.469(9)	C66	C67	1.420(8)
C24	C25	1.417(8)	C66	C69	1.423(9)
C24	C31	1.396(8)	C67	C68	1.354(10)
C25	C26	1.446(9)	C69	C70	1.385(9)
C25	C28	1.371(10)	C70	C72	1.381(9)
C26	C27	1.359(10)	C71	C72	1.374(9)
C28	C29	1.377(10)	C73	C74	1.378(9)
C29	C30	1.379(10)	C73	C78	1.439(8)

Table S4. Bond Lengths for **14a.**

Atom	Atom	Length/Å	Atom	Atom	Length/Å
C30	C31	1.371(9)	C74	C75	1.381(10)
C32	C33	1.374(9)	C75	C76	1.360(10)
C33	C34	1.412(8)	C76	C77	1.426(9)
C34	C35	1.436(8)	C77	C78	1.424(9)
C34	C39	1.432(7)	C77	C82	1.390(9)
C35	C36	1.344(9)	C78	C79	1.430(8)
C36	C37	1.434(8)	C79	C80	1.401(8)
C37	C38	1.438(8)	C80	C81	1.416(9)
C37	C43	1.388(9)	C80	C83	1.432(8)
C38	C39	1.442(8)	C81	C82	1.394(9)
C38	C40	1.406(8)	C81	C86	1.446(8)
C40	C41	1.366(9)	C83	C84	1.365(8)
C41	C42	1.412(9)	C84	C85	1.398(11)
C42	C43	1.355(9)	C85	C86	1.345(10)

Table S5. Bond Angles for **14a.**

Atom	Atom	Atom	Angle/˚	Atom	Atom	Atom	Angle/˚
C2	C1	C6	119.5(6)	C45	C44	C53	121.4(6)
C10	C1	C2	121.3(6)	C49	C44	C45	118.6(6)
C10	C1	C6	119.2(5)	C49	C44	C53	119.9(5)
C3	C2	C1	120.3(7)	C46	C45	C44	121.0(6)
C2	C3	C4	120.6(6)	C45	C46	C47	120.7(6)
C5	C4	C3	120.6(6)	C48	C47	C46	118.4(7)
C4	C5	C6	121.4(7)	C47	C48	C49	120.0(7)
C5	C6	C1	117.6(5)	C44	C49	C48	120.7(6)
C7	C6	C1	120.1(6)	C44	C49	C50	120.3(6)
C7	C6	C5	122.3(6)	C48	C49	C50	118.8(6)
C6	C7	C15	119.8(5)	C51	C50	C49	120.7(6)
C8	C7	C6	119.6(6)	C50	C51	C52	121.6(6)
C8	C7	C15	120.6(5)	C53	C52	C51	119.0(6)
C7	C8	C9	120.7(5)	C57	C52	C51	120.4(5)
C7	C8	C14	123.5(6)	C57	C52	C53	120.5(6)
C14	C8	C9	115.8(6)	C52	C53	C44	116.8(5)
C10	C9	C8	118.8(6)	C54	C53	C44	126.4(5)
C10	C9	C11	121.3(6)	C54	C53	C52	116.8(6)
C11	C9	C8	119.9(6)	C53	C54	C55	118.5(5)
C9	C10	C1	121.7(6)	C53	C54	C64	136.2(6)

Table S5. Bond Angles for **14a.**

Atom	Atom	Atom	Angle/˚	Atom	Atom	Atom	Angle/˚
C12	C11	C9	120.0(7)	C55	C54	C64	105.1(5)
C11	C12	C13	121.2(6)	C56	C55	C54	121.4(5)
C14	C13	C12	119.7(6)	C56	C55	C58	127.8(6)
C13	C14	C8	123.4(7)	C58	C55	C54	110.8(5)
C16	C15	C7	128.0(5)	C57	C56	C55	118.9(6)
C16	C15	C19	105.7(5)	C56	C57	C52	122.0(6)
C19	C15	C7	126.3(5)	C55	C58	C59	106.8(5)
C15	C16	C17	111.9(5)	C55	C58	C79	128.5(5)
C32	C16	C15	126.2(6)	C59	C58	C79	124.7(5)
C32	C16	C17	121.9(5)	C60	C59	C58	128.1(6)
C16	C17	C18	104.2(5)	C60	C59	C64	122.5(5)
C39	C17	C16	118.9(5)	C64	C59	C58	109.4(5)
C39	C17	C18	136.7(6)	C61	C60	C59	119.7(6)
C19	C18	C17	106.3(5)	C60	C61	C62	120.5(5)
C23	C18	C17	134.9(6)	C61	C62	C63	120.8(5)
C23	C18	C19	118.5(5)	C68	C62	C61	119.4(5)
C18	C19	C15	110.7(5)	C68	C62	C63	119.7(6)
C20	C19	C15	127.5(6)	C62	C63	C65	116.9(5)
C20	C19	C18	121.9(6)	C64	C63	C62	116.8(5)
C21	C20	C19	118.0(6)	C64	C63	C65	126.3(5)
C20	C21	C22	121.5(5)	C59	C64	C54	106.7(5)
C21	C22	C23	121.4(6)	C59	C64	C63	116.9(5)
C21	C22	C27	119.3(6)	C63	C64	C54	136.1(5)
C27	C22	C23	119.3(7)	C66	C65	C63	119.2(5)
C18	C23	C22	116.2(6)	C66	C65	C71	118.3(6)
C18	C23	C24	126.1(5)	C71	C65	C63	122.3(5)
C22	C23	C24	117.6(5)	C65	C66	C67	121.7(6)
C25	C24	C23	119.2(5)	C65	C66	C69	119.2(5)
C31	C24	C23	121.9(5)	C67	C66	C69	119.0(6)
C31	C24	C25	118.7(6)	C68	C67	C66	118.9(6)
C24	C25	C26	119.4(6)	C67	C68	C62	122.4(5)
C28	C25	C24	118.7(6)	C70	C69	C66	120.4(6)
C28	C25	C26	121.8(6)	C72	C70	C69	120.0(6)
C27	C26	C25	120.5(6)	C72	C71	C65	121.6(6)
C26	C27	C22	122.1(6)	C71	C72	C70	119.9(6)
C25	C28	C29	122.2(7)	C74	C73	C78	118.9(7)
C28	C29	C30	118.8(7)	C73	C74	C75	122.4(6)
C31	C30	C29	120.8(7)	C76	C75	C74	120.2(6)

Table S5. Bond Angles for **14a.**

Atom	Atom	Atom	Angle/˚	Atom	Atom	Atom	Angle/˚
C30	C31	C24	120.5(6)	C75	C76	C77	121.1(7)
C33	C32	C16	117.8(6)	C78	C77	C76	118.7(6)
C32	C33	C34	120.6(5)	C82	C77	C76	121.9(7)
C33	C34	C35	117.3(5)	C82	C77	C78	119.4(6)
C33	C34	C39	122.2(5)	C77	C78	C73	118.8(6)
C39	C34	C35	120.5(6)	C77	C78	C79	119.7(5)
C36	C35	C34	120.7(5)	C79	C78	C73	121.5(6)
C35	C36	C37	121.9(6)	C78	C79	C58	120.0(5)
C36	C37	C38	118.2(6)	C80	C79	C58	120.8(5)
C43	C37	C36	121.8(6)	C80	C79	C78	119.2(6)
C43	C37	C38	119.9(5)	C79	C80	C81	120.7(5)
C37	C38	C39	120.4(5)	C79	C80	C83	121.8(6)
C40	C38	C37	116.6(5)	C81	C80	C83	117.4(5)
C40	C38	C39	122.9(5)	C80	C81	C86	119.5(6)
C17	C39	C34	116.2(5)	C82	C81	C80	119.3(5)
C17	C39	C38	126.5(5)	C82	C81	C86	121.2(7)
C34	C39	C38	117.3(5)	C77	C82	C81	121.7(6)
C41	C40	C38	121.5(6)	C84	C83	C80	121.2(6)
C40	C41	C42	120.9(6)	C83	C84	C85	120.7(6)
C43	C42	C41	118.6(7)	C86	C85	C84	121.0(6)
C42	C43	C37	122.1(6)	C85	C86	C81	120.2(7)

Table S6. Torsion Angles for **14a.**

A	B	C	D	Angle/˚	A	B	C	D	Angle/˚
C1	C2	C3	C4	0.2(9)	C44	C45	C46	C47	-0.5(10)
C1	C6	C7	C8	1.0(8)	C44	C49	C50	C51	1.9(9)
C1	C6	C7	C15	-177.3(5)	C44	C53	C54	C55	162.9(5)
C2	C1	C6	C5	1.1(8)	C44	C53	C54	C64	-11.4(10)
C2	C1	C6	C7	178.9(5)	C45	C44	C49	C48	6.8(9)
C2	C1	C10	C9	179.7(5)	C45	C44	C49	C50	-167.5(5)
C2	C3	C4	C5	0.3(9)	C45	C44	C53	C52	160.3(5)
C3	C4	C5	C6	-0.1(9)	C45	C44	C53	C54	-18.8(9)
C4	C5	C6	C1	-0.6(8)	C45	C46	C47	C48	6.3(11)
C4	C5	C6	C7	-178.4(5)	C46	C47	C48	C49	-5.5(11)
C5	C6	C7	C8	178.7(5)	C47	C48	C49	C44	-1.0(11)
C5	C6	C7	C15	0.4(8)	C47	C48	C49	C50	173.4(6)
C6	C1	C2	C3	-1.0(8)	C48	C49	C50	C51	-172.5(6)

Table S6. Torsion Angles for **14a.**

A	B	C	D	Angle/˚	A	B	C	D	Angle/˚
C6	C1	C10	C9	-1.6(8)	C49	C44	C45	C46	-6.1(8)
C6	C7	C8	C9	-1.0(8)	C49	C44	C53	C52	-15.5(8)
C6	C7	C8	C14	176.3(5)	C49	C44	C53	C54	165.3(5)
C6	C7	C15	C16	111.8(6)	C49	C50	C51	C52	-4.5(10)
C6	C7	C15	C19	-66.4(7)	C50	C51	C52	C53	-3.3(8)
C7	C8	C9	C10	-0.3(8)	C50	C51	C52	C57	179.8(6)
C7	C8	C9	C11	179.8(5)	C51	C52	C53	C44	13.0(7)
C7	C8	C14	C13	-178.7(6)	C51	C52	C53	C54	-167.8(5)
C7	C15	C16	C17	178.0(5)	C51	C52	C57	C56	179.6(5)
C7	C15	C16	C32	-3.9(9)	C52	C53	C54	C55	-16.2(7)
C7	C15	C19	C18	174.2(5)	C52	C53	C54	C64	169.4(5)
C7	C15	C19	C20	-5.2(9)	C53	C44	C45	C46	178.1(6)
C8	C7	C15	C16	-66.5(8)	C53	C44	C49	C48	-177.3(6)
C8	C7	C15	C19	115.3(6)	C53	C44	C49	C50	8.4(9)
C8	C9	C10	C1	1.6(8)	C53	C52	C57	C56	2.7(9)
C8	C9	C11	C12	-2.0(9)	C53	C54	C55	C56	12.7(8)
C9	C8	C14	C13	-1.2(9)	C53	C54	C55	C58	-166.7(4)
C9	C11	C12	C13	0.6(9)	C53	C54	C64	C59	163.5(6)
C10	C1	C2	C3	177.7(5)	C53	C54	C64	C63	-22.8(10)
C10	C1	C6	C5	-177.5(5)	C54	C55	C56	C57	-0.8(8)
C10	C1	C6	C7	0.2(8)	C54	C55	C58	C59	-3.5(6)
C10	C9	C11	C12	178.1(6)	C54	C55	C58	C79	177.3(5)
C11	C9	C10	C1	-178.5(5)	C55	C54	C64	C59	-11.4(5)
C11	C12	C13	C14	0.5(10)	C55	C54	C64	C63	162.3(6)
C12	C13	C14	C8	-0.1(10)	C55	C56	C57	C52	-6.9(9)
C14	C8	C9	C10	-177.9(5)	C55	C58	C59	C60	173.1(5)
C14	C8	C9	C11	2.2(8)	C55	C58	C59	C64	-4.1(6)
C15	C7	C8	C9	177.3(5)	C55	C58	C79	C78	115.2(6)
C15	C7	C8	C14	-5.4(8)	C55	C58	C79	C80	-67.2(7)
C15	C16	C17	C18	9.2(6)	C56	C55	C58	C59	177.1(5)
C15	C16	C17	C39	-165.7(4)	C56	C55	C58	C79	-2.1(9)
C15	C16	C32	C33	178.7(5)	C57	C52	C53	C44	-170.1(5)
C15	C19	C20	C21	177.6(5)	C57	C52	C53	C54	9.1(8)
C16	C15	C19	C18	-4.3(6)	C58	C55	C56	C57	178.5(5)
C16	C15	C19	C20	176.2(5)	C58	C59	C60	C61	178.9(5)
C16	C17	C18	C19	-11.4(5)	C58	C59	C64	C54	9.6(5)
C16	C17	C18	C23	161.9(6)	C58	C59	C64	C63	-165.4(4)
C16	C17	C39	C34	-17.1(7)	C58	C79	C80	C81	-175.6(5)

Table S6. Torsion Angles for **14a.**

A	B	C	D	Angle/˚	A	B	C	D	Angle/˚
C16	C17	C39	C38	160.4(5)	C58	C79	C80	C83	0.8(8)
C16	C32	C33	C34	-7.3(8)	C59	C58	C79	C78	-63.9(7)
C17	C16	C32	C33	-3.5(8)	C59	C58	C79	C80	113.7(6)
C17	C18	C19	C15	9.9(6)	C59	C60	C61	C62	-5.9(8)
C17	C18	C19	C20	-170.6(5)	C60	C59	C64	C54	-167.7(5)
C17	C18	C23	C22	168.8(5)	C60	C59	C64	C63	17.2(7)
C17	C18	C23	C24	-14.8(9)	C60	C61	C62	C63	2.8(8)
C18	C17	C39	C34	170.1(5)	C60	C61	C62	C68	-174.2(5)
C18	C17	C39	C38	-12.4(10)	C61	C62	C63	C64	10.2(7)
C18	C19	C20	C21	-1.8(8)	C61	C62	C63	C65	-167.8(5)
C18	C23	C24	C25	169.8(5)	C61	C62	C68	C67	177.0(5)
C18	C23	C24	C31	-16.3(8)	C62	C63	C64	C54	167.5(5)
C19	C15	C16	C17	-3.5(6)	C62	C63	C64	C59	-19.3(7)
C19	C15	C16	C32	174.6(5)	C62	C63	C65	C66	-11.8(7)
C19	C18	C23	C22	-18.6(7)	C62	C63	C65	C71	162.6(5)
C19	C18	C23	C24	157.8(5)	C63	C62	C68	C67	0.0(8)
C19	C20	C21	C22	-6.7(9)	C63	C65	C66	C67	5.6(8)
C20	C21	C22	C23	2.1(9)	C63	C65	C66	C69	-177.2(5)
C20	C21	C22	C27	-178.2(5)	C63	C65	C71	C72	178.9(5)
C21	C22	C23	C18	10.8(8)	C64	C54	C55	C56	-171.4(5)
C21	C22	C23	C24	-165.9(5)	C64	C54	C55	C58	9.2(6)
C21	C22	C27	C26	176.3(6)	C64	C59	C60	C61	-4.3(8)
C22	C23	C24	C25	-13.8(7)	C64	C63	C65	C66	170.4(5)
C22	C23	C24	C31	160.0(5)	C64	C63	C65	C71	-15.1(8)
C23	C18	C19	C15	-164.7(4)	C65	C63	C64	C54	-14.7(10)
C23	C18	C19	C20	14.8(8)	C65	C63	C64	C59	158.4(5)
C23	C22	C27	C26	-3.9(9)	C65	C66	C67	C68	3.8(8)
C23	C24	C25	C26	3.2(8)	C65	C66	C69	C70	-4.0(8)
C23	C24	C25	C28	179.2(6)	C65	C71	C72	C70	0.7(9)
C23	C24	C31	C30	-178.5(6)	C66	C65	C71	C72	-6.7(8)
C24	C25	C26	C27	7.6(9)	C66	C67	C68	C62	-6.7(8)
C24	C25	C28	C29	-1.5(11)	C66	C69	C70	C72	-2.1(9)
C25	C24	C31	C30	-4.7(8)	C67	C66	C69	C70	173.3(6)
C25	C26	C27	C22	-7.3(10)	C68	C62	C63	C64	-172.8(5)
C25	C28	C29	C30	-2.8(12)	C68	C62	C63	C65	9.2(7)
C26	C25	C28	C29	174.4(7)	C69	C66	C67	C68	-173.4(5)
C27	C22	C23	C18	-169.0(5)	C69	C70	C72	C71	3.7(9)
C27	C22	C23	C24	14.3(7)	C71	C65	C66	C67	-169.1(5)

Table S6. Torsion Angles for **14a.**

A	B	C	D	Angle/˚	A	B	C	D	Angle/˚
C28	C25	C26	C27	-168.3(7)	C71	C65	C66	C69	8.2(8)
C28	C29	C30	C31	3.4(11)	C73	C74	C75	C76	2.1(10)
C29	C30	C31	C24	0.4(10)	C73	C78	C79	C58	-6.1(8)
C31	C24	C25	C26	-170.8(5)	C73	C78	C79	C80	176.3(5)
C31	C24	C25	C28	5.2(9)	C74	C73	C78	C77	-0.6(8)
C32	C16	C17	C18	-168.9(5)	C74	C73	C78	C79	-179.0(5)
C32	C16	C17	C39	16.2(8)	C74	C75	C76	C77	-0.8(9)
C32	C33	C34	C35	-173.6(5)	C75	C76	C77	C78	-1.1(8)
C32	C33	C34	C39	5.6(8)	C75	C76	C77	C82	177.7(6)
C33	C34	C35	C36	178.9(5)	C76	C77	C78	C73	1.7(8)
C33	C34	C39	C17	6.9(7)	C76	C77	C78	C79	-179.8(5)
C33	C34	C39	C38	-170.8(5)	C76	C77	C82	C81	-179.3(5)
C34	C35	C36	C37	-4.4(9)	C77	C78	C79	C58	175.5(5)
C35	C34	C39	C17	-173.9(5)	C77	C78	C79	C80	-2.1(8)
C35	C34	C39	C38	8.3(7)	C78	C73	C74	C75	-1.3(9)
C35	C36	C37	C38	0.7(8)	C78	C77	C82	C81	-0.5(8)
C35	C36	C37	C43	-174.7(6)	C78	C79	C80	C81	2.0(8)
C36	C37	C38	C39	7.7(8)	C78	C79	C80	C83	178.4(5)
C36	C37	C38	C40	-168.0(5)	C79	C58	C59	C60	-7.7(9)
C36	C37	C43	C42	171.3(6)	C79	C58	C59	C64	175.2(5)
C37	C38	C39	C17	170.6(5)	C79	C80	C81	C82	-1.2(8)
C37	C38	C39	C34	-12.0(7)	C79	C80	C81	C86	177.8(5)
C37	C38	C40	C41	-5.4(8)	C79	C80	C83	C84	-177.5(5)
C38	C37	C43	C42	-4.0(9)	C80	C81	C82	C77	0.4(8)
C38	C40	C41	C42	-0.3(9)	C80	C81	C86	C85	0.0(9)
C39	C17	C18	C19	162.2(6)	C80	C83	C84	C85	-0.7(9)
C39	C17	C18	C23	-24.6(10)	C81	C80	C83	C84	-1.0(8)
C39	C34	C35	C36	-0.3(8)	C82	C77	C78	C73	-177.1(5)
C39	C38	C40	C41	179.0(5)	C82	C77	C78	C79	1.4(8)
C40	C38	C39	C17	-14.0(8)	C82	C81	C86	C85	179.0(6)
C40	C38	C39	C34	163.4(5)	C83	C80	C81	C82	-177.7(5)
C40	C41	C42	C43	4.0(10)	C83	C80	C81	C86	1.3(8)
C41	C42	C43	C37	-1.8(10)	C83	C84	C85	C86	2.1(10)
C43	C37	C38	C39	-176.8(5)	C84	C85	C86	C81	-1.8(9)
C43	C37	C38	C40	7.5(8)	C86	C81	C82	C77	-178.6(5)

Table S7. Hydrogen Atom Coordinates (Å×10^4) and Isotropic Displacement Parameters (Å^2×10^3) for **14a**.

**Atom**	***x***	***y***	***z***	**U(eq)**
H2	4408.92	4807	6435.73	52
H3	3656.73	3689.8	6031.26	55
H4	3936.89	3732.85	5346.39	53
H5	4971.81	4881.83	5064.98	50
H10	5463.17	6266.94	6509.04	50
H11	6549.12	7687.06	6595.53	55
H12	7631.96	8821.69	6323.59	58
H13	7988.12	8855.23	5640.25	58
H14	7255.89	7770.64	5237.75	52
H20	7445.36	4836.65	5136.64	51
H21	8311.74	3935.38	4665.8	49
H26	8269.99	3590.47	3334.57	58
H27	8592.29	3462.98	3997.72	52
H28	6984.33	4102.45	2807.05	68
H29	5544.14	4996.7	2586.72	71
H30	4612.33	5925.37	3034.41	61
H31	5210.27	6062.67	3677.68	44
H32	4714.45	7851.5	5057.9	47
H33	4118.41	8803.59	4546.25	45
H35	4362.55	9458.16	3901.54	47
H36	5069.87	9424.27	3282.07	49
H40	7710.17	6740.01	3744.17	43
H41	8567.53	6881.71	3145.37	55
H42	7853.86	7858.41	2650.79	57
H43	6392.17	8784.79	2798.23	53
H45	7026.66	1120.41	6523.83	45
H46	7485.06	1188.09	7182.63	60
H47	6372.6	441.06	7666.52	69
H48	4999.75	-581.89	7448.37	64
H50	3955.26	-1288.63	6914.07	54
H51	3683.61	-1551.08	6262.18	50
H56	4856.94	-317.81	5081.48	47
H57	4004.5	-1152.2	5562.2	50
H60	7552.05	2722.54	5086.43	45
H61	8152.7	3738.4	5559.84	47
H67	7239.69	4471.44	6836.76	50
H68	7943.51	4435.44	6209.97	48

Table S7. Hydrogen Atom Coordinates (Å×10[4]) and Isotropic Displacement Parameters (Å[2]×10[3]) for **14a**.

**Atom**	***x***	***y***	***z***	**U(eq)**
H69	5834.82	3964.22	7307.07	51
H70	4323.28	3061.72	7460.04	58
H71	4519.87	1825.74	6401.03	45
H72	3623.63	2043.47	6992.81	51
H73	4966.19	2661.26	4963.74	49
H74	4216.05	3762.99	4550.35	57
H75	4584.18	3788.73	3881.85	60
H76	5633.56	2654.6	3599.29	57
H82	6706.5	1207.29	3666.43	51
H83	7273.83	-233.45	5087.87	46
H84	8317.21	-1348.73	4788.98	54
H85	8539.17	-1368.24	4105.08	56
H86	7818.23	-228.45	3720.69	52

Table S8. Solvent masks information for **14a**.

**Number**	**X**	**Y**	**Z**	**Volume**	**Electron count**	**Content**
1	-0.726	0.250	0.258	397.5	94.3	?
2	0.193	0.750	0.758	397.5	94.3	?

Experimental

Single crystals of $C_{43}H_{25}$ **14a** were grown by freezing its dry toluene solution at -20 °C. A suitable crystal was selected and performed on a **SuperNova, Dual, Cu at zero, AtlasS2** diffractometer. The crystal was kept at 179.99(10) K during data collection.

4. DFT Calculations:

Computational Methods:

(U)B3LYP /6-31G(d,p)

All of the DFT calculations were performed with Gaussian 16 software packages[3]. The optimization calculations and single-point energy calculations were employed at (U)B3LYP[4] level of density functional theory (DFT) the S_0 state and time-dependent density functional theory (TD-DFT) of the excited states, using the 6-31G (d, p) basis sets[5] for all atoms. Vibrational frequency analysis was calculated at the same level of theory to verify whether each optimized structure is an energy minimum and to evaluate its zero-point vibrational energy. All

of the product structures were fully optimized without any symmetric restrictions. Intrinsic reaction coordinate (IRC) calculations[6] were conducted to confirm that the transition state is connected to the correct reactant and product. 3D structures were generated by CYLview[7]. The weak interaction IRI[8] analyses and the spin density analyses were performed by Multiwfn[9] to obtain the wavefunctions from the geometry. The weak interaction and the spin density visualized by VMD.[10]

Table S9. Thermal correction of Gibbs free energy (TCG, hartree), single-point energies (E, hartree), E+ Thermal Free Energy Correction (G, hartree) and E + Thermal Enthalpies Correction (H, hartree)

Compounds	TCG	E	G	H
**14a**	0.476145	-1653.713374	-1653.237229	-1653.146014
**TS**	0.478460	-1653.675185	-1653.196724	-1653.109160
**folder**	0.476494	-1653.678083	-1653.201589	-1653.111063
**$(14a)_2$**	0.982881	-3307.422775	-3306.439893	-3306.281992
**15a**	0.464207	-1464.387950	-1463.923744	-1463.831510

14a

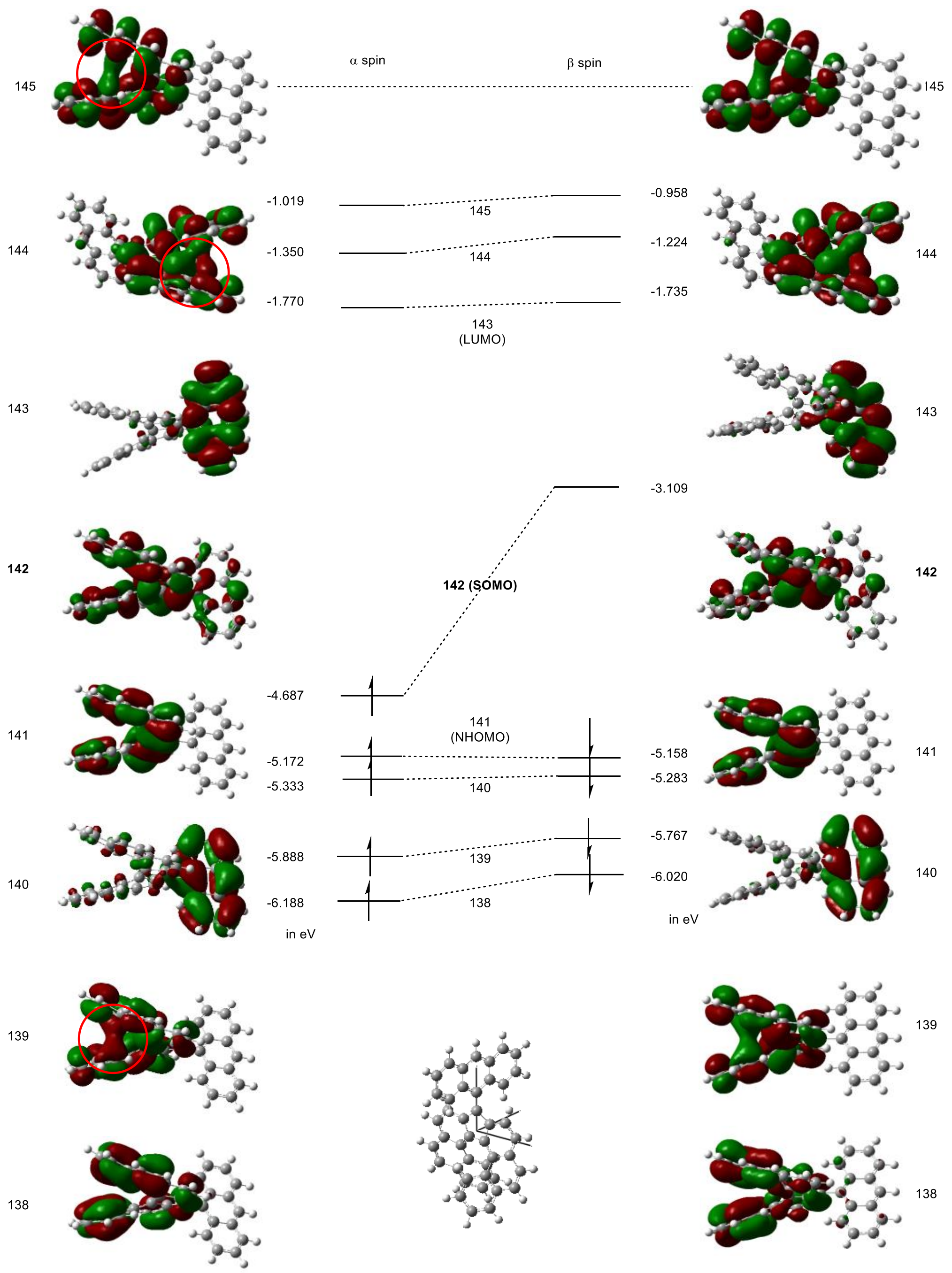

Table S10. Excited states, excitation energies and oscillator strengths of **14a.**

****

Excited states from <AA,BB:AA,BB> singles matrix:

****

Ground to excited state transition electric dipole moments (Au):

state	X	Y	Z	Dip. S.	Osc.
1	0.0000	-0.1629	0.1421	0.0467	0.0013
2	-0.9882	0.0000	-0.0000	0.9764	0.0357
3	-0.8537	0.0000	-0.0000	0.7288	0.0330
4	0.0000	-0.8152	0.3738	0.8043	0.0382
5	0.7323	-0.0000	0.0000	0.5362	0.0292

Excitation energies and oscillator strengths:

Excited State 1: 2.083-A 1.1147 eV 1112.22 nm f=0.0013 <S**2>=0.835

141B ->142B 0.97845

Excited State 2: 2.361-A 1.4909 eV 831.58 nm f=0.0357 <S**2>=1.143

140A ->143A 0.26250

142A ->143A 0.21350

140B ->142B 0.90312

140B ->143B -0.23829

Excited State 3: 3.091-A 1.8505 eV 669.99 nm f=0.0330 <S**2>=2.139

140A ->143A -0.51481

142A ->143A -0.41176

134B ->149B 0.10203

140B ->142B 0.41943

140B ->143B 0.59116

140B <-143B 0.11001

Excited State 4: 2.077-A 1.9384 eV 639.62 nm f=0.0382 <S**2>=0.829

141A ->144A 0.10636

142A ->145A 0.11060

139B ->142B 0.97319

Excited State 5: 2.164-A 2.2246 eV 557.33 nm f=0.0292 <S**2>=0.920

141A ->145A 0.16921

142A ->144A -0.19507

142A ->146A -0.14005

137B ->142B 0.54004

138B ->142B 0.75553

****

15a

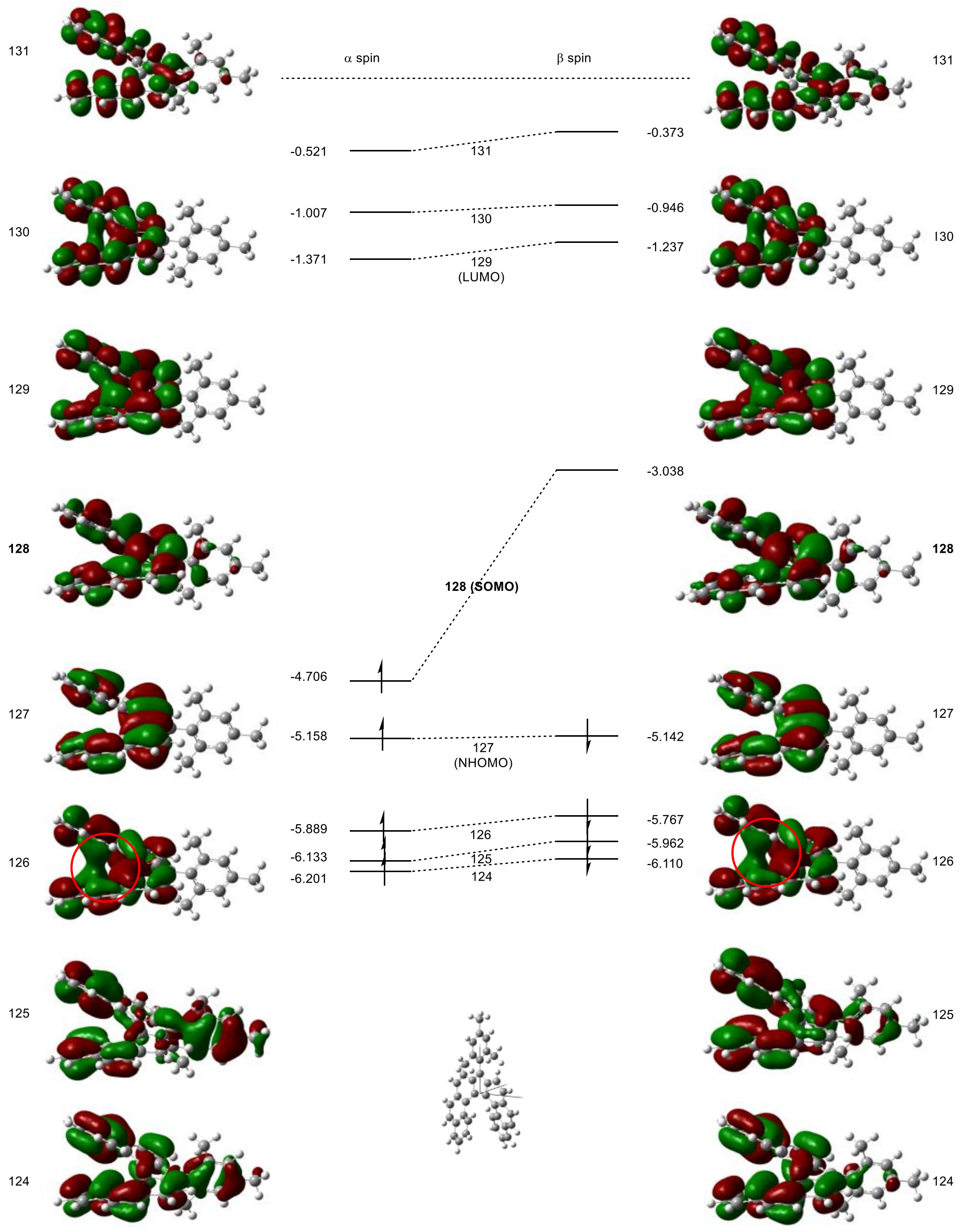

Table S11. Excited states, excitation energies and oscillator strengths of **15a**

****

Excited states from <AA,BB:AA,BB> singles matrix:

****

Ground to excited state transition electric dipole moments (Au):

state	X	Y	Z	Dip. S.	Osc.
1	0.0001	0.1773	0.1091	0.0433	0.0012
2	0.0008	0.8505	0.2108	0.7678	0.0374
3	-1.0846	-0.0002	0.0000	1.1764	0.0645
4	-0.0187	0.0002	-0.0000	0.0004	0.0000
5	0.0165	-0.0013	0.0000	0.0003	

Excitation energies and oscillator strengths:

Excited State 1: 2.089-A 1.1433 eV 1084.46 nm f=0.0012 <S**2>=0.841

121B ->128B 0.11098

127B ->128B 0.97849

Excited State 2: 2.083-A 1.9865 eV 624.13 nm f=0.0374 <S**2>=0.835

127A ->129A -0.11826

128A ->130A 0.12212

126B ->128B 0.97149

Excited State 3: 2.125-A 2.2370 eV 554.23 nm f=0.0645 <S**2>=0.879

127A ->130A 0.15201

128A ->131A 0.16060

125B ->128B 0.95614

Excited State 4: 2.126-A 2.2693 eV 546.35 nm f=0.0000 <S**2>=0.880

127A ->130A 0.10784

128A ->129A 0.31894

124B ->128B 0.91874

Excited State 5: 2.286-A 2.4730 eV 501.35 nm f=0.0000 <S**2>=1.057

127A ->130A -0.19946

128A ->129A 0.70844

123B ->128B -0.56659

124B ->128B -0.21475

127B ->130B -0.10034

****

Table S12. Cartesian coordinates for optimized geometries

14a

C	-0.62306700	-0.74365100	-0.03033200
C	-0.62310200	0.74367800	0.03033800
C	0.74493800	1.14825900	-0.10781800
C	1.59471800	0.00007300	0.00001700
C	0.74498000	-1.14815700	0.10786500
C	1.11328700	-2.49981600	0.25443900
C	0.12280000	-3.45365600	0.26117200
C	-1.21957000	-3.10985500	-0.03783500
C	-1.59395700	-1.74656400	-0.31543100
C	-1.59403900	1.74654500	0.31539600
C	-1.21973100	3.10984700	0.03772500
C	0.12262200	3.45372000	-0.26123200
C	1.11317200	2.49993600	-0.25439300
C	-2.21065300	-4.14203000	-0.08009100
C	-3.48935600	-3.88774300	-0.46920400
C	-3.85545100	-2.59160900	-0.95792100
C	-2.90183700	-1.52742400	-0.92973200
C	-2.90190000	1.52737000	0.92972700
C	-3.85558000	2.59149600	0.95784000

C	-3.48957700	3.88761400	0.46899900
C	-2.21089100	4.14195700	0.07987600
C	3.07101400	0.00007200	0.00002000
C	3.78215900	-0.32197800	1.18655600
C	5.22672000	-0.32586200	1.17652600
C	5.90792000	0.00001200	0.00003400
C	5.22674300	0.32589900	-1.17647200
C	3.78217500	0.32207200	-1.18653500
C	3.12428500	-0.61221400	2.42443600
C	3.83688900	-0.91577500	3.55540200
C	5.25996300	-0.94199700	3.53421200
C	5.93134800	-0.65040400	2.37778500
C	5.93141500	0.65039300	-2.37771600
C	5.26007400	0.94199800	-3.53417000
C	3.83700700	0.91580700	-3.55540000
C	3.12436000	0.61228300	-2.42444700
H	2.15597100	-2.76746600	0.38997100
H	0.36148200	-4.49796800	0.44144900
H	0.36125600	4.49803200	-0.44157200
H	2.15584000	2.76767000	-0.38987100
H	-1.91136500	-5.14609800	0.20831600
H	-4.23410100	-4.67892100	-0.48205800
H	-4.23437600	4.67874000	0.48179300
H	-1.91166900	5.14601700	-0.20863000
H	6.99547100	0.00001900	0.00004100
H	2.04146000	-0.58416900	2.45939800
H	3.31210700	-1.13205100	4.48126500
H	5.80716500	-1.18510600	4.44013000
H	7.01784000	-0.65475000	2.35116400
H	7.01790500	0.65471200	-2.35106300
H	5.80731000	1.18511000	-4.44006600

H	3.31225100	1.13204800	-4.48128700
H	2.04154000	0.58421300	-2.45947500
C	-3.24239300	0.33039200	1.59910900
H	-2.50861600	-0.46025800	1.67388100
C	-5.12675900	2.37975900	1.53778600
H	-5.84331300	3.19707900	1.52896100
C	-4.48168300	0.15574600	2.18983300
H	-4.70521800	-0.77691700	2.69896100
C	-5.44553300	1.17705400	2.13788000
H	-6.42236100	1.03113200	2.58942400
C	-3.24242500	-0.33039800	-1.59898200
H	-2.50870400	0.46031300	-1.67366700
C	-5.12666000	-2.37989300	-1.53781400
H	-5.84316400	-3.19725600	-1.52904500
C	-5.44552000	-1.17715100	-2.13778900
H	-6.42237200	-1.03124200	-2.58928500
C	-4.48173700	-0.15577500	-2.18966500
H	-4.70534100	0.77691800	-2.69870700

TS

C	-0.57135000	-0.57881900	-0.30058700
C	-0.65561200	0.87248900	-0.04336300
C	0.65147100	1.41607000	-0.24173100
C	1.62476000	0.31209600	-0.12309900
C	0.80559000	-0.91432200	-0.23407500
C	1.22427400	-2.24488900	-0.43904500
C	0.27886100	-3.22964500	-0.60357400
C	-1.09400200	-2.91785000	-0.73619100
C	-1.52842500	-1.55225600	-0.73094700
C	-1.71088300	1.72296900	0.40342600
C	-1.55866900	3.12598400	0.16181300

C	-0.33688700	3.62101500	-0.34344500
C	0.76584200	2.80465000	-0.46989700
C	-2.04247100	-3.97653500	-0.91498000
C	-3.35226800	-3.71726800	-1.16912100
C	-3.79144300	-2.37295900	-1.39713700
C	-2.87453600	-1.28644400	-1.23770300
C	-2.89468300	1.28571500	1.13805300
C	-3.95082400	2.21757200	1.38196200
C	-3.81269100	3.58015500	0.95963200
C	-2.64863200	4.02089700	0.41242000
C	3.02720600	0.25234300	0.12892800
C	3.53943300	-0.89667300	0.87750000
C	4.82643100	-1.44937500	0.56880800
C	5.64312100	-0.78454300	-0.36379600
C	5.33578400	0.53243800	-0.75798200
C	4.07774400	1.13681000	-0.38438800
C	2.82223400	-1.45655100	1.96137500
C	3.29987900	-2.55573000	2.65047000
C	4.51717500	-3.16394400	2.27084900
C	5.26552600	-2.61886300	1.25169800
C	6.30865200	1.31144600	-1.44630800
C	6.13310200	2.66080100	-1.65145600
C	4.98160400	3.28636900	-1.13391300
C	3.98867600	2.53779800	-0.52439200
H	2.27344400	-2.50478600	-0.45015500
H	0.58443700	-4.26866700	-0.69082100
H	-0.25999600	4.67624600	-0.58986000
H	1.68053800	3.23540600	-0.83834600
H	-1.68445000	-4.99965300	-0.83685400
H	-4.06950200	-4.52595800	-1.28077300
H	-4.63704200	4.26533200	1.13824900

H	-2.51900000	5.06880500	0.15543000
H	6.58757600	-1.22680300	-0.66908700
H	1.88434500	-1.00107400	2.25753800
H	2.73627100	-2.95174500	3.48962800
H	4.87441300	-4.04074700	2.80264400
H	6.22657800	-3.04738600	0.98042400
H	7.22518500	0.82059700	-1.76249200
H	6.89483300	3.24536000	-2.15803000
H	4.87624600	4.36550600	-1.19398800
H	3.16294900	3.05343300	-0.06424800
C	-3.01212800	0.00095600	1.71717100
H	-2.19708200	-0.70276600	1.61596600
C	-5.10023800	1.80541800	2.09426400
H	-5.89782400	2.52674200	2.25277800
C	-4.13610000	-0.37258000	2.43155200
H	-4.18945500	-1.36748200	2.86351300
C	-5.20223200	0.52725200	2.60621900
H	-6.08749600	0.22608300	3.15847100
C	-3.29533200	-0.01633300	-1.69549000
H	-2.59607600	0.80723900	-1.68948500
C	-5.10192300	-2.12616400	-1.86695800
H	-5.78631100	-2.96608400	-1.95523900
C	-5.49714800	-0.85955700	-2.24795100
H	-6.50295100	-0.68570300	-2.61885600
C	-4.57036100	0.19550600	-2.18867400
H	-4.85078900	1.18541500	-2.53604400

Folder

C	-0.62704200	-0.77467300	-0.18041000
C	-0.62483600	0.70629500	-0.12121800
C	0.69872800	1.13096200	-0.39824700

C	1.62138500	-0.00366100	-0.14322400
C	0.73031600	-1.19240700	-0.12615800
C	1.05136000	-2.55977000	-0.19021500
C	0.03945900	-3.49424300	-0.17524000
C	-1.31485100	-3.11102300	-0.27118600
C	-1.66222400	-1.73074300	-0.43579500
C	-1.62319300	1.69102600	0.15564000
C	-1.38017300	3.01027800	-0.34351800
C	-0.13484700	3.32478200	-0.93520100
C	0.91408800	2.43118400	-0.89606700
C	-2.33733300	-4.11653400	-0.24674300
C	-3.63964500	-3.80418000	-0.47235600
C	-4.00280800	-2.48029500	-0.88448900
C	-3.01108100	-1.45056300	-0.93038100
C	-2.82049000	1.47426200	0.95824300
C	-3.80620200	2.50587200	1.03861400
C	-3.58483900	3.75602500	0.37406000
C	-2.40594900	4.00737800	-0.25527300
C	2.98703200	0.04133500	0.10996100
C	3.78242400	-1.13758500	0.51807800
C	5.05978700	-1.33416900	-0.10083800
C	5.62434500	-0.28464600	-0.87004000
C	5.11780300	1.03175600	-0.71923700
C	3.84770000	1.23241700	-0.08071800
C	3.39073400	-2.02455800	1.53278500
C	4.15956800	-3.13878600	1.85281500
C	5.34418200	-3.40821800	1.14491100
C	5.78831400	-2.51759800	0.18497500
C	5.91058100	2.16174700	-1.04730200
C	5.54385500	3.42960300	-0.63575700
C	4.37846400	3.60115000	0.13035200

C	3.54553300	2.51743800	0.39371500
H	2.07669100	-2.89192700	-0.23652100
H	0.27824700	-4.55388600	-0.14577100
H	0.00335800	4.30856900	-1.37485100
H	1.87787700	2.71888200	-1.29024800
H	-2.03941200	-5.14028100	-0.03654000
H	-4.41251600	-4.56681300	-0.42827900
H	-4.35741700	4.51822100	0.43060800
H	-2.21333200	4.97932900	-0.70171500
H	6.57027900	-0.44049100	-1.38123600
H	2.46665900	-1.83682400	2.06805700
H	3.83853400	-3.80323900	2.64914800
H	5.92749900	-4.29408800	1.37759100
H	6.73542500	-2.68163300	-0.32191300
H	6.84790200	2.00365300	-1.57391700
H	6.17576900	4.28219600	-0.86567500
H	4.12238300	4.58165000	0.52007500
H	2.64793300	2.66586700	0.98167500
C	-3.01425900	0.31979500	1.75256900
H	-2.25099200	-0.44698300	1.76824100
C	-4.96681200	2.30671300	1.82127000
H	-5.71089200	3.09842000	1.85579000
C	-4.14723900	0.15701700	2.52822300
H	-4.26183800	-0.74139900	3.12739700
C	-5.14504100	1.14826100	2.55060700
H	-6.03754900	1.01166500	3.15409900
C	-3.36624500	-0.24426800	-1.57671000
H	-2.61392100	0.51562000	-1.73081900
C	-5.31451600	-2.21681200	-1.34068700
H	-6.05556400	-3.00893600	-1.26987900
C	-5.64235600	-1.00145100	-1.90783100

H	-6.65041100	-0.81623300	-2.26689700
C	-4.64543300	-0.02207900	-2.05490600
H	-4.87418100	0.91645800	-2.55085800

$(\mathbf{14a})_2$

C	-3.66829900	0.33677500	1.07961100
C	-4.55500200	1.34887500	0.44917700
C	-4.28177800	2.63295900	-0.05828900
H	-3.26886500	3.00414600	-0.11496600
C	-5.32129200	3.40620000	-0.53099800
H	-5.11905300	4.37275900	-0.98401200
C	-6.66482900	2.99897900	-0.36896200
C	-7.72964300	3.88117100	-0.74934100
H	-7.46696000	4.82600300	-1.21763900
C	-9.03053600	3.55583100	-0.52808100
H	-9.83004200	4.22827700	-0.82736700
C	-9.37256200	2.35510500	0.17561500
C	-8.34524300	1.43808600	0.55815800
C	-6.96546800	1.70780500	0.17059800
C	-5.86190500	0.81562100	0.35082300
C	-5.74720200	-0.65523200	0.50887200
C	-6.58684500	-1.74257000	0.10453700
C	-7.69429300	-1.67697000	-0.84849700
C	-8.58232200	-2.79127000	-0.96933200
C	-8.31955100	-3.99672400	-0.23946700
H	-9.01792500	-4.82413100	-0.33191300
C	-7.17021000	-4.13089100	0.47111600
H	-6.91776100	-5.07484700	0.94668200
C	-6.25450400	-3.03641600	0.62136700
C	-5.01195500	-3.25099200	1.25325400
H	-4.76385500	-4.25564600	1.58468900

C	-4.09350100	-2.23203200	1.39365000
H	-3.11294100	-2.45330200	1.78587100
C	-4.46373800	-0.91957800	1.05265800
C	-2.41508800	0.53449800	1.59839400
C	-1.61304200	1.79137600	1.49407200
C	-2.04588500	3.03557000	1.97168300
H	-3.04249500	3.12881400	2.38969900
C	-1.20421300	4.14594800	1.91836300
H	-1.55144300	5.10327700	2.29556400
C	0.08345000	4.02141300	1.39469400
H	0.74181100	4.88393900	1.35114500
C	0.53998800	2.77586200	0.96118900
H	1.55679300	2.66846100	0.59356300
C	-0.28760000	1.65309300	1.02844500
C	0.22211800	0.25459900	0.71508800
H	1.31545200	0.27111400	0.72359100
C	-0.26048200	-0.65178700	1.83652900
C	0.58641300	-1.55613400	2.48033400
H	1.60483800	-1.68032400	2.12258500
C	0.14623300	-2.27165600	3.59488300
H	0.81942900	-2.96095200	4.09583800
C	-1.14604600	-2.07238200	4.08354800
H	-1.48168300	-2.59749900	4.97286800
C	-2.00674300	-1.18656200	3.43638600
H	-3.00897800	-1.02237000	3.81889300
C	-1.58739500	-0.49736800	2.29065000
C	3.66820900	-0.33670400	-1.07953000
C	4.55486800	-1.34879000	-0.44901100
C	4.28155500	-2.63276600	0.05868100
H	3.26861400	-3.00386500	0.11543300
C	5.32101200	-3.40599300	0.53153800

H	5.11870300	-4.37245000	0.98473600
C	6.66457700	-2.99888300	0.36946500
C	7.72932500	-3.88105800	0.75006300
H	7.46656800	-4.82578300	1.21853600
C	9.03024700	-3.55583300	0.52880800
H	9.82969900	-4.22826600	0.82826700
C	9.37237700	-2.35525900	-0.17509400
C	8.34512600	-1.43825900	-0.55786700
C	6.96531400	-1.70783000	-0.17033200
C	5.86181100	-0.81561300	-0.35076500
C	5.74721300	0.65522200	-0.50903800
C	6.58699100	1.74252600	-0.10492500
C	7.69461200	1.67686800	0.84789400
C	8.58271900	2.79111700	0.96857900
C	8.31987800	3.99658600	0.23876000
H	9.01831400	4.82395500	0.33107100
C	7.17043000	4.13080300	-0.47164400
H	6.91795900	5.07476600	-0.94718600
C	6.25464000	3.03637500	-0.62174200
C	5.01201700	3.25098900	-1.25347300
H	4.76391900	4.25564400	-1.58490500
C	4.09350300	2.23206000	-1.39373500
H	3.11290300	2.45335600	-1.78584400
C	4.46371000	0.91960800	-1.05270600
C	2.41497600	-0.53438800	-1.59827600
C	1.61286300	-1.79122000	-1.49390400
C	2.04563300	-3.03544900	-1.97149300
H	3.04223100	-3.12875600	-2.38952100
C	1.20390300	-4.14578200	-1.91813200
H	1.55107800	-5.10313800	-2.29531400
C	-0.08374400	-4.02116800	-1.39444600

H	-0.74215000	-4.88365800	-1.35086400
C	-0.54021300	-2.77558000	-0.96097000
H	-1.55700600	-2.66811800	-0.59333000
C	0.28743200	-1.65285600	-1.02827000
C	-0.22221900	-0.25433000	-0.71495300
H	-1.31555400	-0.27079800	-0.72346400
C	0.26042900	0.65201200	-1.83641100
C	-0.58641900	1.55639500	-2.48022700
H	-1.60483700	1.68064400	-2.12247800
C	-0.14620200	2.27188400	-3.59478300
H	-0.81936200	2.96121200	-4.09574400
C	1.14606900	2.07254400	-4.08344300
H	1.48173500	2.59764000	-4.97276400
C	2.00671900	1.18668300	-3.43627400
H	3.00894900	1.02244300	-3.81877500
C	1.58733000	0.49751100	-2.29054000
C	-7.87053400	-0.60955900	-1.75909800
H	-7.15760600	0.20249900	-1.76848800
C	-9.66402800	-2.73467600	-1.87753800
H	-10.33863400	-3.58523000	-1.93248900
C	-8.91390300	-0.59178700	-2.66723800
H	-9.01048500	0.24421200	-3.35346500
C	-9.84076700	-1.64747100	-2.70989800
H	-10.66872000	-1.62179800	-3.41217700
C	-8.71244200	0.35702100	1.39348300
H	-7.94623600	-0.31013500	1.76492200
C	-10.71243000	2.10387500	0.55067400
H	-11.47955400	2.80585000	0.23391500
C	-11.04264400	1.01195200	1.32803500
H	-12.07463000	0.83568300	1.61669500
C	-10.02620200	0.14575100	1.76932100

H	-10.27034200	-0.69293900	2.41448000
C	8.71242800	-0.35737600	-1.39338600
H	7.94627500	0.30974400	-1.76499700
C	10.71227800	-2.10417100	-0.55013100
H	11.47934900	-2.80612700	-0.23320200
C	11.04259100	-1.01241800	-1.32768600
H	12.07460100	-0.83626200	-1.61632900
C	10.02621800	-0.14624600	-1.76919200
H	10.27044000	0.69230700	-2.41449900
C	7.87100500	0.60938400	1.75838200
H	7.15806300	-0.20266500	1.76783600
C	9.66459600	2.73444000	1.87657600
H	10.33925100	3.58496200	1.93143300
C	8.91456100	0.59152100	2.66630300
H	9.01125600	-0.24451600	3.35246700
C	9.84144600	1.64719400	2.70886100
H	10.66952600	1.62146300	3.41098800

15a

C	0.12000300	-0.73904600	-0.09461000
C	0.11918800	0.73866000	0.09381900
C	-1.25118100	1.11283800	0.28917200
C	-2.09850900	-0.00136400	-0.00076500
C	-1.24991800	-1.11473300	-0.29024800
C	-1.62256700	-2.41970200	-0.66568900
C	-0.63420300	-3.35990000	-0.84331600
C	0.71040400	-3.07262000	-0.49819100
C	1.08972000	-1.77716600	0.00766400
C	1.08780800	1.77785200	-0.00801200
C	0.70696200	3.07278900	0.49801900
C	-0.63802100	3.35850800	0.84299700

C	-1.62531800	2.41727900	0.66493600
C	1.70053100	-4.09687100	-0.64130500
C	2.98214100	-3.91441900	-0.22261900
C	3.35343700	-2.72264900	0.48091600
C	2.40138000	-1.66929300	0.64307500
C	2.39977000	1.67149300	-0.64305300
C	3.35063400	2.72586000	-0.48046900
C	2.97784800	3.91711400	0.22314800
C	1.69593600	4.09809100	0.64155300
C	-3.58041700	-0.00187300	-0.00048700
C	-4.28912700	0.09171700	-1.21921900
C	-5.68687100	0.08635800	-1.19461000
C	-6.40579000	-0.00717600	0.00017900
C	-5.68595000	-0.10312000	1.19425800
C	-4.28822200	-0.10073200	1.21839600
H	-2.66890200	-2.65737700	-0.83193800
H	-0.87506900	-4.35757500	-1.19935300
H	-0.88002000	4.35581600	1.19930200
H	-2.67194100	2.65366700	0.83119900
H	1.39799800	-5.03587300	-1.09706100
H	3.72558600	-4.69632900	-0.35180500
H	3.72040500	4.69981300	0.35265700
H	1.39227600	5.03667800	1.09741600
C	2.74637400	0.60927100	-1.50798900
H	2.01435700	-0.15707900	-1.72285000
C	4.62558100	2.61958300	-1.08059400
H	5.34023700	3.42388800	-0.92595900
C	3.98933900	0.54149500	-2.11282200
H	4.21798800	-0.28821000	-2.77488500
C	4.95041900	1.53954200	-1.87838200
H	5.93015700	1.47548500	-2.34250600

C	2.74656900	-0.60658800	1.50798300
H	2.01365200	0.15898900	1.72253600
C	4.62808700	-2.61490700	1.08140800
H	5.34366100	-3.41845800	0.92709600
C	4.95151100	-1.53441500	1.87916200
H	5.93103700	-1.46923900	2.34357700
C	3.98927500	-0.53738600	2.11318900
H	4.21681500	0.29264300	2.77522800
H	-6.22736700	0.16095600	-2.13578400
H	-6.22565800	-0.18871700	2.13494600
C	-7.91590500	0.02162300	0.00311700
H	-8.32556900	-0.54272500	0.84623300
H	-8.29291100	1.04881200	0.08628600
H	-8.32645300	-0.40010600	-0.91923700
C	-3.55824000	0.21517800	-2.53709900
H	-2.88233100	1.07703500	-2.53868500
H	-2.94294600	-0.66682900	-2.74447900
H	-4.26303200	0.33609100	-3.36391100
C	-3.55615100	-0.22944600	2.53511200
H	-2.88014400	-1.09123200	2.53317400
H	-2.94077200	0.65193000	2.74491500
H	-4.26021000	-0.35312500	3.36214000

5. References:

[1] H. Oyama, M. Akiyama, K. Nakano, M. Naito, K. Nobusawa, K. Nozaki, *Org. Lett.* **2016**, *18*, 3654-3657.

[2] J. Feng, L. Wang, X. Xue, Z. Chao, B. Hong, Z. Gu, *Org. Lett.* **2021**, *23*, 8056-8061.

[3] M. J. Frisch, G. W. Trucks, H. B. Schlegel, G. E. Scuseria, M. A. Robb, J. R. Cheeseman, G. Scalmani, V. Barone, G. A. Petersson, H. Nakatsuji, X. Li, M. Caricato, A. V. Marenich, J. Bloino, B. G. Janesko, R. Gomperts, B. Mennucci, H. P. Hratchian, J. V. Ortiz, A. F. Izmaylov, J. L. Sonnenberg, D. Williams-Young, F. Ding, F. Lipparini, F. Egidi, J. Goings, B. Peng, A. Petrone, T. Henderson, D. Ranasinghe, V. G. Zakrzewski, J. Gao, N. Rega, G. Zheng, W. Liang, M. Hada, M. Ehara, K. Toyota, R. Fukuda, J. Hasegawa, M. Ishida, T. Nakajima, Y. Honda, O. Kitao, H. Nakai, T. Vreven, K. Throssell, J. A. Montgomery, Jr., J. E. Peralta, F. Ogliaro, M. J. Bearpark, J. J. Heyd, E. N. Brothers, K. N. Kudin, V. N. Staroverov, T. A. Keith, R. Kobayashi, J. Normand, K. Raghavachari, A. P. Rendell, J. C. Burant, S. S. Iyengar, J. Tomasi, M. Cossi, J. M. Millam, M. Klene, C. Adamo, R. Cammi, J. W. Ochterski, R. L. Martin, K. Morokuma, O. Farkas, J. B. Foresman, and D. J. Fox. *Gaussian 16, RevisionC.03*, Gaussian, Inc., Wallingford CT, **2016**.

[4] (a) B. Miehlich, A. Savin, H. Stoll, and H. Preuss. *Chem. Phys. Lett.*, **1989**, *157*, 200–206. (b) C. Lee, W. Yang, and R. G. Parr. *Phys. Rev. B.,* **1988**, *37*, 785–89.

[5] (a) G. A. Petersson, A. Bennett, T. G. Tensfeldt, M. A. Al-Laham, W. A. Shirley, and J. Mantzaris. *J. Chem. Phys.* **1988***, 89*, 2193–218 (cited on pages 18, 76, 78). (b) G. A. Petersson and M. A. Al-Laham. *J. Chem. Phys.,* **1991**, *94*, 6081–90 (cited on pages 18, 76, 78).

[6] Fukui, K. *Acc. Chem. Res.* **1981**, *14*, 363-368.

[7] CYLview, 1.0b; Legault, C. Y., Université de Sherbrooke, **2009** (http://www.cylview.org).

[8] T. Lu, Q. Chen, *Chemistry—Methods* **2021**, *1*, 231-239.

[9] T. Lu, F. Chen, *J. Comput. Chem.* **2012**, 33, 580-592.

[10] W. Humphrey, A. Dalke, K. Schulten, *J. Molecul.Graph*.**1996**,*14*, 33-38.
